# Combination of transcriptomic and proteomic approaches helps unravel the mechanisms of luteolin in inducing liver cancer cell death via targeting AKT1 and SRC

**DOI:** 10.3389/fphar.2024.1450847

**Published:** 2024-08-21

**Authors:** Junxia Ma, Jinggang Mo, Yifu Feng, Liezhi Wang, Hao Jiang, Junmin Li, Chong Jin

**Affiliations:** ^1^ Department of General Surgery, Taizhou Central Hospital (Taizhou University Hospital), Taizhou University, Taizhou, Zhejiang, China; ^2^ Zhejiang Provincial Key Laboratory of Evolutionary Ecology and Conservation, Taizhou University, Taizhou, China

**Keywords:** traditional Chinese medicine, luteolin, transcriptomics, proteomics, liver cancer

## Abstract

**Introduction:**

Luteolin, a natural compound commonly used in traditional Chinese medicine, shows clinical potential as an anti–liver cancer agent. The mechanisms underlying the anti–liver cancer effect of luteolin are limited *versus* those reported for other cancers. Accordingly, this study was conducted to bridge the existing knowledge gap.

**Methods:**

Transcriptomic and proteomic analyses of the response of the hepatocellular carcinoma cell line HuH-7 to luteolin were conducted, and a possible pathway was elucidated using confocal laser scanning microscopy (CLSM), flow cytometry, western blotting, qRT-PCR and bio-layer interferometry assay to systematically explore the possible mechanisms underlying the inhibition of the proliferation of liver cancer cells by luteolin.

**Results and Discussion:**

Results showed that luteolin significantly inhibited HuH-7 cell proliferation. Transcriptomic and proteomic analyses collectively revealed that luteolin could promote cell cycle arrest and apoptosis in HuH-7 cells through transcription factors p53, nuclear factor kappa B (NF-κB), FOXO, ATF2, and TCF/LEF via AKT1, as well as the KEAP-NRF and SRC-STAT3 pathways. Furthermore, AKT1 and SRC were identified as the 2 targets of luteolin. Nuclear translocation of transcription factors p53 and NF-κB were affected by luteolin administration. Additionally, AKT1 activity affected normal metabolism in HuH-7 cells and resulted in the accumulation of reactive oxygen species, which activated MOMP and further promoted apoptosis. Our results systematically elucidate the mechanism of luteolin in inhibiting the proliferation of liver cancer cells, mainly through cell cycle arrest and apoptosis via targeting AKT1 and SRC.

## 1 Introduction

For centuries, the plant kingdom has been an excellent source of natural compounds in the form of herbal extracts to treat benign and malignant neoplasms (Salehi et al., 2020). More than 10,000 flavonoids have been identified among the bioactive compounds from plants (Ullah et al., 2020; Slika et al., 2022). Understanding the effect of plant-derived flavonoids in treating cancer and elucidating their potential mechanisms of action could promote the development of novel and reliable phytochemicals (Imran et al., 2019).

Luteolin (3,4′,5,7-tetrahydroxyflavone), one of the most extensively researched flavonoids, is a flavone that is widely present in many plant species such as carrots, celery, onion leaves, broccoli, parsley, sweet bell peppers, chrysanthemum flowers, and *Cyclocarya paliurus* (Imran et al., 2019; Caporali et al., 2022; Mo et al., 2023). Luteolin exerts multiple biological effects such as antiallergy, anti-inflammatory, antidiabetic, neuroprotective, antioxidant, and anticancer effects (Muruganathan et al., 2022). The anticancer properties of luteolin have been demonstrated in various cancer types such as colon, lung, prostate, gastric, and breast cancers as well as glioblastoma (Imran et al., 2019; Ganai et al., 2021; Çetinkaya and Baran, 2023). Liver cancer is more common in males and is the fifth cause of cancer-related deaths in males (Ganai et al., 2021). Luteolin can inhibit the proliferation of liver cancer cells in a dose- and time-dependent manner but has no effect on normal liver cells (Ganai et al., 2021), indicating its potential as an anticancer agent.

Various mechanistic events of the anticancer activity of luteolin have been well documented in different cancers (Imran et al., 2019; Ganai et al., 2021) including liver cancer (Ding et al., 2014; Yee et al., 2015) (for review, see Supplementary Table S1). Yee et al. (2015) found that luteolin could inhibit HepG2 cell proliferation by inducing apoptosis, causing G1 cell cycle arrest and upregulating the expression of p21WAF1/CIP1, transforming growth factor Smad4 β1 (TGF-β1), p27KIP1, and Fas. Ding et al. (2014) have reported that luteolin inhibits the proliferation of liver carcinoma cells (SMMC-7721 and BEL-7402) by arresting the cell cycle at the G1/S phase by increasing Bax and caspase-3 expression and reducing the levels of the anti-apoptotic protein Bcl-2. However, the mechanisms underlying the anti–liver cancer effect of luteolin are limited *versus* those reported for other cancers.

Recently, based on network pharmacology and molecular docking, we found that luteolin could suppress liver cancer cell proliferation by promoting apoptosis and via cell cycle arrest through various signaling pathways related to the Ser/Thr/Tyr residue binding site–containing proteins such as MAP2K1, EGFR, PIK3CA, MET, SRC, and AKT1 (Mo et al., 2023). To systematically explore the possible mechanisms underlying the antiproliferative effect of luteolin on liver cancer cells, an approach combining transcriptomic analysis, proteomic analysis, and computational biology was chosen throughout the process of luteolin treatment in the hepatocellular carcinoma cell line HuH-7. Abnormal and proportional gene expression related to the Ser/Thr/Tyr residue binding site–containing proteins, including SRC, AKT1, MET, phosphatidylinositol-4,5-bisphosphate 3-kinase catalytic subunit α (PIK3CA), MAP2K1, MAPK3, and MAP3K7 were noted in HuH-7 cells (Kasai et al., 2018; Ji et al., 2023). The possible pathway was further studied using immunofluorescence staining. Our findings could help gain global insights into the biological and physiological processes mediated by a single flavonoid compound in treating liver cancer. The results not only provide a high-quality proteogenomic resource of a luteolin-treated hepatocellular carcinoma cell line but also imply promising prognostic and therapeutic significance and underlying regulatory mechanisms that may benefit clinical practice.

## 2 Materials and methods

### 2.1 Samples and cells

Luteolin was purchased from Chengdu Desite Biotechnology Co. LTD (Chengdu, China). HuH-7 cells were purchased from the National Collection of Authenticated Cell Cultures, Shanghai City, China. High-glucose DMEM medium supplemented with 10% fetal bovine serum (FBS) (Univ, Shanghai, China) and 1% penicillin/streptomycin (Sangon Biotech, Shanghai, China) was used to culture the cells in a humidified atmosphere of 95% air and 5% CO_2_ at 37°C in a CO_2_ incubator (MEMMERT, Germany). Media was replaced at 2-day intervals.

### 2.2 *In vitro* effects of luteolin on cell activity and the proliferation of HuH-7 cells

Cells were cultured according to the method described by Mo et al. (2023). Cell activity was assessed using a 3-(4,5-dimethylthiazol-2-yl)-2,5-diphenyltetrazolium bromide (MTT) cell proliferation and cytotoxicity assay kit (Universal Biotech Co., Ltd, Shanghai, China) (Mo et al., 2023).

HuH-7 cells were cultured in 4-well plates (Xinyou Technology Co., Ltd., Hangzhou, China) at 2.5 × 10^4^ cells per mL for 24 h, treated with 20 µM luteolin dissolved in DMSO, and incubated for 24 h and 48 h. Paclitaxel (10 µM) dissolved in DMSO was used as the positive control. For the cell count assay, HuH-7 cells were treated as described above. Next, the cells were digested using trypsin and counted using CLSM (IX83-FV3000, Olympus Corporation, Tokyo, Japan) using the differential interference contrast module. Three wells from each group were chosen and the average number of cells was calculated.

### 2.3 Cell sample collection

HuH-7 cells were cultured in 10-cm dishes for 24 h at a density of 2.5 × 10^4^ cells per mL. Next, they were treated with 20 µM luteolin dissolved in DMSO and incubated for 24 h and 48 h. An equal volume of DMSO with 0 µM luteolin was used as the negative control. Next, the cells were trypsinized, washed three times with phosphate-buffered saline (PBS), collected into 1.5-mL RNase/DNase-free tubes, and centrifuged for 5 min at 1,200 rpm. The supernatant was discarded and the 1.5-mL tubes with the cell precipitates were quickly frozen in liquid nitrogen. A total of such 4 groups, namely, Luteonlin_24, Control_24, Luteolin_48, and Control_48, were used for subsequent transcriptomic and proteomic analyses, with three biological replicates for each group.

### 2.4 Transcriptomics analysis

#### 2.4.1 RNA extraction and sequencing

Total RNA was extracted from HuH-7 cells using an RNA Easy Fast Tissue/Cell kit (Sangon Biotech Co., Ltd., Shanghai, China) according to the manufacturer’s instructions. RNA integrity was assessed using an RNA Nano 6000 assay kit and a Bioanalyzer 2,100 system (Agilent Technologies, Santa Clara, CA, United States). mRNA was enriched using Oligo (dT) magnetic beads. Next, mRNA was fragmented randomly using the fragmentation buffer. The first and second cDNA strands were synthesized in the m-MulLV reverse transcriptase system and DNA polymerase I system, respectively. cDNA of approximately 370–420 bp was screened for amplification using polymerase chain reaction (PCR), and the PCR products were purified using AMPure XP Beads to obtain the library. A Qubit2.0 fluorometer was used for initial quantification after library construction. The library was diluted to 1.5 ng/μL, and Agilent 2,100 BioAnalyzer was used to measure the insert size of the library. Illumina sequencing was performed after pooling the different libraries according to the requirements of effective concentration and target onboard data amount, and 150 bp paired-end raw data were generated.

#### 2.4.2 *De novo* assembly and annotation

Raw data were filtered by removing the reads with adapters, reads containing undetermined base N, and poor-quality reads to ensure the quality and reliability of data analysis. All subsequent analyses were performed using clean data. Trinity (version 2.0.6) (Trinity Technologies, Irvine, CA, United States) was used for the *de novo* assembly of clean reads from the obtained samples (Grabherr et al., 2011). Transcripts were assembled and gene function was annotated based on the following databases: NCBI nonredundant protein sequence (Nr), NCBI nonredundant nucleotide sequence (Nt), protein family (Pfam), clusters of orthologous groups of proteins (KOG/COG), SwissProt (a manually annotated and reviewed protein sequence database), Kyoto Encyclopedia of Genes and Genomes (KEGG) Ortholog (KO) database, and Gene Ontology (GO). GO functional annotation was obtained by comparing the transcripts with the SwissProt and TrEMBL databases, and KEGG transcript annotation information was based on KAAS acquisition.

#### 2.4.3 Data analysis

Analysis of differentially expressed genes (DEGs) was performed for two conditions/groups using DESeq2 R package (1.20.0). Genes with an adjusted *p*-value <0.05 and a |log_2_FC| > 1 (FC, fold change) as determined using DESeq2 were designated as differentially expressed. Principal component analysis (PCA) was performed using the gplots package in R to identify sample clusters and distribution patterns. For cluster analysis, a heatmap was generated based on the analysis results using Tbtools software (GitHub, Inc., San Francisco, CA, United States).

### 2.5 Proteomics analysis

#### 2.5.1 Total protein extraction

Total protein from cells was extracted following a previous method with some modifications (Wiśniewski et al., 2009; Kachuk et al., 2015; Satpathy et al., 2020). Protein quality was determined using 12% sodium dodecyl sulfate–polyacrylamide gel electrophoresis and Coomassie brilliant blue R-250 staining. Bovine serum albumin (BSA) was used as the standard protein.

#### 2.5.2 Lysing of proteins

The sample was transferred to a 1.5-mL centrifuge tube and lysed with DB lysis buffer (8 M urea, 100 mM triethylammonium bicarbonate; pH 8.5), followed by 5 min of ultrasonication on ice. The lysate was centrifuged at 12,000 × g for 15 min at 4°C, and the supernatant was added with 1 M DL-dithiothreitol to react for 1 h at 56°C and subsequently alkylated with sufficient iodoacetamide for 1 h at room temperature in the dark followed by reaction in an ice-bath for 2 min.

#### 2.5.3 Trypsin treatment

The volume of each protein sample was made up to 100 μL with DB lysis buffer (8 M urea, 100 mM TEAB; pH 8.5). Trypsin and 100 mM TEAB buffer were added and the samples were mixed and digested for 4 h at 37°C. Next, trypsin and CaCl2 were added and digested overnight. Formic acid was added to the digested sample, the pH was adjusted to <3, and the sample was centrifuged at 12,000 g for 5 min at room temperature. The supernatant was slowly loaded to a C18 desalting column and washed 3 times with the washing buffer (0.1% formic acid, 3% acetonitrile). Elution buffer was added (0.1% formic acid, 70% acetonitrile) and the eluents of each sample were collected and lyophilized.

#### 2.5.4 Separation of fractions (high-depth quantification)

Mobile phases A (2% acetonitrile, pH adjusted to 10.0 using ammonium hydroxide) and B (98% acetonitrile, pH adjusted to 10.0 using ammonium hydroxide) were used for gradient elution. The lyophilized powder was dissolved in solution A and centrifuged at 12,000 × g for 10 min at room temperature. The sample was fractionated using a C18 column (Waters BEH C18, 4.6 × 250 mm, 5 μm) using a Rigol L3000 high-performance liquid chromatography (HPLC) system, and the column oven was set at 45°C. Details of the elution gradient are as follows: 97%:3% (mobile phase A:B) at 0 min; 95%:5% at 10 min; 80%:20% at 30 min; 60%:40% at 48 min; 50%:50% at 50 min; 30%:70% at 53 min; 0%:100% at 54 min. The eluates were monitored at 214 nm using UV. One tube of eluent was collected per minute and subsequently combined into 10 fractions. All fractions were dried under vacuum and reconstituted in 0.1% (v/v) formic acid in water (Zhang et al., 2016).

#### 2.5.5 Liquid chromatography–mass spectrometry (LC-MS)/MS

The separated peptides were analyzed using a Q Exactive TM HF-X mass spectrometer with Nanospray Flex™ (ESI) as an ion source, spray voltage of 2.1 kV, and ion transport capillary temperature of 320°C. Full scan range from m/z 350 to 1,500 with a resolution of 60,000 (at m/z 200), automatic gain control (AGC) target value of 3 × 106, and maximum ion injection time of 20 m were used as the parameters. The top 40 precursors of the highest abundance in the full scan were selected and fragmented using higher energy collision dissociation and analyzed in MS/MS, where the resolution was 15,000 (at m/z 200), AGC target value was 1 × 105, and maximum ion injection time was 45 m. Normalized collision energy was set as 27%, the intensity threshold was 2.2 × 104, and the dynamic exclusion parameter was 20 s.

#### 2.5.6 Data analysis

##### 2.5.6.1 Identification and quantitation of proteins

The acquired spectra were searched against the Gallus_gallus_uniprot_2021_7_15. fasta.fata database using the Proteome Discoverer (Thermo, HFX and 480) or MaxQuant (Bruker, Tims) search engines. The search parameters for Proteome Discoverer were set as follows: mass tolerance for the precursor ion: 10 ppm, mass tolerance for the product ion: 0.02 Da. Carbamidomethyl was specified as fixed modifications, oxidation of methionine (M) was specified as dynamic modification and loss of methionine at the N-terminal. A maximum of two missed cleavage sites were allowed. The search parameters of MaxQuant were set as follows: mass tolerance for the precursor ion: 20 ppm, mass tolerance for product ion: 0.05 Da. Carbamidomethyl was specified as fixed modifications, oxidation of methionine (M) was specified as dynamic modification, and acetylation was specified as N-terminal modification. A maximum of two missed cleavage sites were allowed.

To improve the quality of analysis results, the software PD or MaxQuant was used to further filter the retrieval results. Peptide spectrum matches (PSMs) with a credibility >99% were considered. The identified proteins contained at least one unique peptide. The identified PSMs and proteins were retained and performed with a false discovery rate (FDR) of ≤1.0%. Protein quantitation results were statistically analyzed using *t*-test. Proteins for which the quantitation was significantly different between the experimental and control groups (-log_10_ *p*-value <0.05 and |log2FC| > 0.6) were defined as differentially expressed proteins (DEPs).

##### 2.5.6.2 Functional analysis of proteins and DEPs

GO and InterPro (IPR) functional analyses were conducted using the InterProScan program against the nonredundant protein database (including Pfam, PRINTS, ProDom, SMART, ProSite, and PANTHER) (Jones et al., 2014), and the Clusters of Orthologous Groups and KEGG databases were used to analyze the protein families and pathways. DEPs were used for volcanic map analysis, cluster heatmap analysis, and enrichment analysis of GO, IPR, and KEGG (Huang et al., 2009). The probable protein–protein interactions were predicted using the STRING-db server (Franceschini et al., 2012) (http://string.embl.de/).

### 2.6 Association analysis of mRNA and proteins

Spearman correlation coefficient was used to determine the correlation between mRNA expression and protein abundance for each gene–protein pair across all 24 HuH-7–luteolin samples. In addition, *p* values corresponding to the correlation coefficient were computed and adjusted using the FDR correction. Significance of the correlation pair was determined based on an adjusted *p*-value cutoff of 0.01.

### 2.7 Cell apoptosis

HuH-7 cells were cultured in 4-well plates, treated, and grouped to determine apoptosis using CLSM (IX83-FV3000, Olympus Corporation, Tokyo, Japan) as described in Section 2.3. The cells were washed 3 times with PBS and stained using an Apoptotic and Necrotic Detection Kit Triple Fluorescence dye (Sangon Biotech, Shanghai, China) (Mo et al., 2023).

HuH-7 cells were cultured in 24-well plates, treated, and grouped as described in Section 2.3 to determine apoptosis using flow cytometry (BD FACS Aria II, New Jersey, United States). The cells were harvested, centrifuged for 5 min at 300 × g, washed 3 times with PBS, and stained using the Apoptotic and Necrotic Detection Kit Triple Fluorescence dye (Mo et al., 2023).

### 2.8 Immunofluorescence staining for p53 and nuclear factor kappa B

HuH-7 cells were cultured in 4-well plates (Xinyou Technology Co., Ltd., Hangzhou, China) at a density of 2.5 × 10^4^ cells per mL. They were then treated with 20 µM luteolin dissolved in DMSO and incubated for 24 h. An equal volume of DMSO containing 0 µM luteolin was used as the negative control. For immunofluorescence staining, the culture medium was aspirated and the cells were washed 3 times with cold PBS. Next, the cells were fixed in 4% paraformaldehyde and permeabilized using 0.1% Triton X-100. Subsequently, the cells were blocked with QuickBlock™ (P0260, Beyotime Biotechnology, Shanghai, China) and incubated with antibodies against NF-κB p65 (AF1234, Beyotime Biotechnology, Shanghai, China) and p53 (AF0255, Beyotime Biotechnology, Shanghai, China). After washing in PBS 3 times, the cells were incubated with FITC-conjugated secondary antibodies (A0562 and A0568, Beyotime Biotechnology, Shanghai, China). DAPI and DiI were used to stain the nuclei and membranes, respectively (C991 S and C1005, Beyotime Biotechnology, Shanghai, China). Lastly, the cells were visualized using CLSM (IX83-FV3000, Olympus, Japan).

### 2.9 Combination of luteolin with the target protein

To determine the combination of luteolin with the target protein AKT1 and SRC, luteolin was dissolved in DMSO to prepare a 10 mM stock solution and was then diluted with PBS to final concentrations of 20, 10, 5 and 2.5 µM for AKT1, and 20, 10, 5 and 2.5 µM for SRC for further analysis. The combination of luteolin with AKT1 was tested using bio-layer interferometry (BLI) according to the manufacturer’s instructions and as described by Wang et al. (2024). First, we performed buffer exchange of 0.5 mL AKT1 (50 μg/mL, 01–101, Carna Biosciences, Inc.) using desalting columns included in the biotinylation kit (G-MM-IGT, Genemore). Second, we added 1 µL of biotin reagent to 0.1 mL of AKT1 solution obtained in the first step, mixed the reaction mixture well, and incubated it at room temperature for 30–60 min. Third, we performed buffer exchange as the first step and finally obtained approximately 0.5 mL of biotinylated AKT1 (about 50 μg/mL). Next, biotinylated AKT1 was loaded on a Super Streptavidin Biosensor (SSA) (18–5,057, Sartorius AG, Goettingen, Germany) obtained from Sartorius. The SSA sensor was equilibrated in PBS and loaded with AKT1 solution containing 50 μg/mL AKT1 for 10 min. Reference SSA sensors were set up by blocking them with assay buffer (PBS +0.1% BSA +0.02% Tween 20 + 5% DMSO) for 10 min. SSA sensors were loaded to a shift of up to 4 nm with biotinylated AKT1 on a ForteBio Octet K2 Biolayer Interferometer (Sartorius AG, Goettingen, Germany). Lastly, test plates were prepared by adding 200 µL of the assay buffer in 96-well plates (Greiner). The assay settings were as follows: custom time: 600 s; baseline time: 60 s; association time: 90 s; dissociation time: 120 s; shake speed: 1,000 rpm. The resulting data were processed using the double-reference method using ForteBio Octet Analysis software (Sartorius AG, Goettingen, Germany).

The combination of luteolin with SRC (50 μg/mL, UA080128, UA Bioscience, Inc.) was tested as above with AKT1.

### 2.10 qRT-PCR confirmation of RNA-Seq data

Seven candidate genes relating to ROS, cell cycle arrest and cell apoptosis were chosen and RT-qPCR was conducted to verify gene expression. The primer sequences are shown in Supplementary Table S3. Real-time PCR was performed as the procedure described by Wang et al. (2024).

### 2.11 Statistical analysis

Results are presented as the mean ± standard deviation. Statistical analysis was performed using SPSS 19.0. One-way analysis of variance was used to determine the significance of comparisons between groups after confirming the homogeneity of variance. Fisher’s least-significant difference test was used for comparative analysis between the control group and treatment groups.

## 3 Results

### 3.1 *In vitro* effects of luteolin on HuH-7 cell proliferation

**FIGURE 1:**
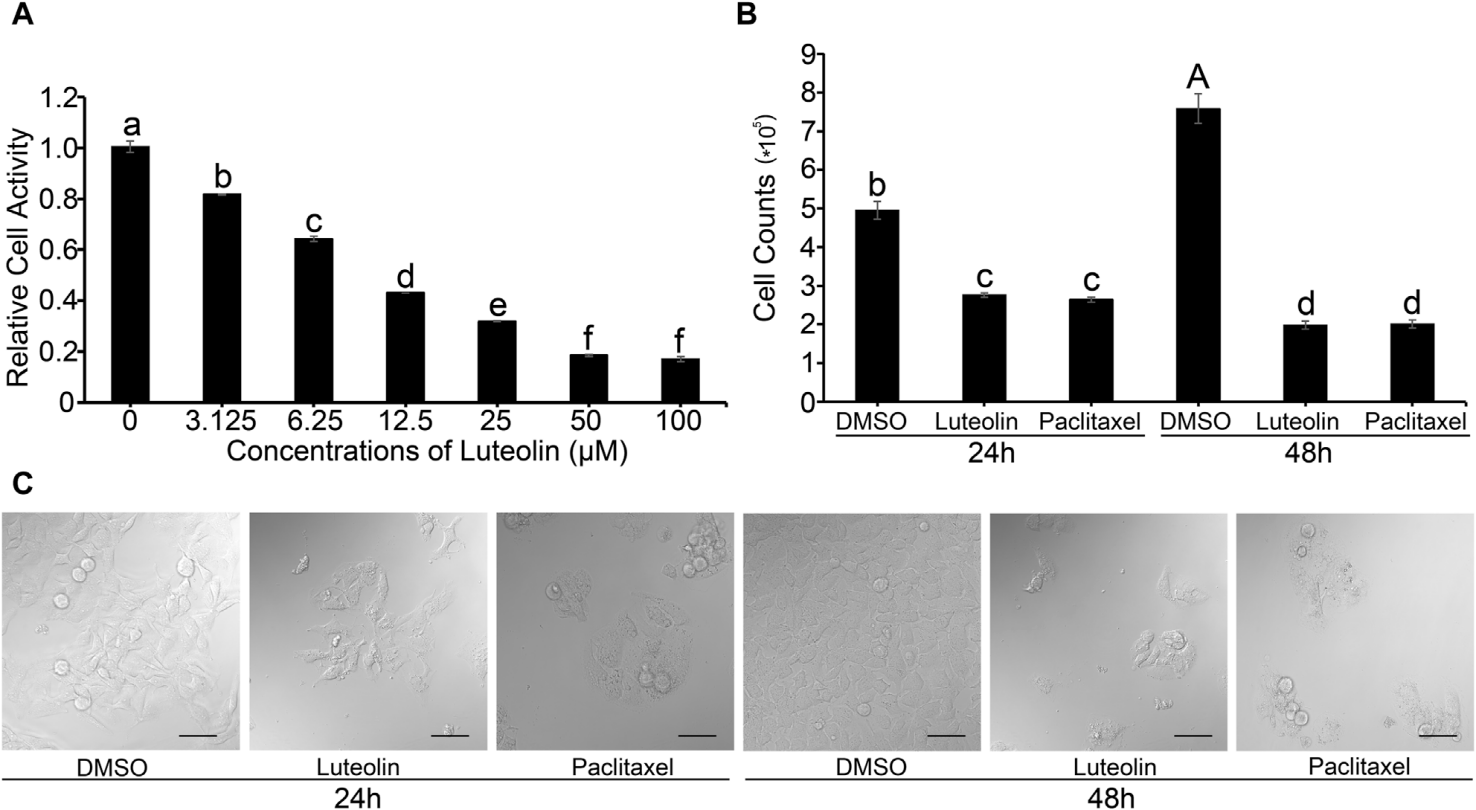
Effects of luteolin on HuH-7 cells. **(A)** Relative cell activity. **(B)** Cell count. Data are expressed as the mean ± STDEV (n = 3). Uppercase (*p* < 0.01) and lowercase (*p* < 0.05) letters indicate significant differences between two treatments. **(C)** Representative CLSM images of HuH-7 cells treated with 20 µM luteolin and 10 µM paclitaxel for 24 or 48 h, respectively. Data are representative of four independent experiments. Bars = 50 µm.

The MTT assay revealed that the proliferation activity of HuH-7 cells decreased in a concentration-dependent manner as luteolin concentration increased (Figure 1A). When the concentration of luteolin was 50 μM, the proliferation activity of HuH-7 cells was only 16.7% relative to the DMSO-only control (Figure 1A). The half-maximal inhibitory concentration (IC_50_) of luteolin on the proliferation activity of HuH-7 cells was 12.68 µM. Luteolin significantly inhibited cell proliferation in HuH-7 cells (Figures 1B, C). The cell proliferation and survival rate both decreased significantly at 48 h compared to that at 24 h (Figures 1B, C). These results showed that luteolin inhibited the proliferation of HuH-7 cells *in vivo* in a concentration-dependent manner.

### 3.2 Overview of transcriptomic and proteomic data

#### 3.2.1 The sequencing quality analysis

RNA integrity was assessed using the RNA Nano 6000 Assay Kit of the Bioanalyzer 2,100 system (Agilent Technologies, CA, USA), with a 150 bp read length. A total of 1,076, 554, 280 reads of sequencing data, including 553, 237, 046 raw reads and 523, 317, 234 clean reads were obtained. The average Q20 and Q30 values, and GC content were 97.08% and 92.26%, and 49.92%, respectively (Supplementary Table S1).

Each mass spectrometer has its own measurement range, so there is a limit to the length of the peptides that can be identified, and peptides that are too long or too short to be detected in the mass spectrometer. If the peptides are generally too short or too long in the identification, it may be that the protease has been inappropriately selected. The distribution of the peptide length range is shown in Supplementary Figure S1. There is a certain deviation between the molecular weight of the precursor ion (primary mass spectrometry, i.e., peptide ions) measured by mass spectrometry and the theoretical molecular weight of the peptide, which is an inherent property of the mass spectrometer, and an important indicator to measure the performance of mass spectrometry, and can also be used as a reference for the quality of the identification results. The mass deviation distribution of the measured and theoretical molecular weights of the peptide precursor ion is shown in Supplementary Figure S2. The identified peptides and proteins are obtained through protein database comparison, and the proteins containing exactly the same peptides are called the proteins of the same group, and the unique peptides in each group are called Unique peptides, which make the protein group have unique specificity, and the more unique peptides, the more reliable the identified proteins. The distribution of the number of unique peptides is shown in Supplementary Figure S3. The greater the number of peptides that support an identified protein, the more confidence the protein is indicated. Therefore, the identification coverage of the protein can also indirectly reflect the overall accuracy of the identification results. The protein coverage distribution is shown in Supplementary Figure S4. Protein molecular weight distribution is an important indicator to assess the size of the identified protein. The wider the molecular weight range, the wider the range of proteins identified. Protein molecular weight distribution is shown in Supplementary Figure S5.

#### 3.2.2 Identification and functional enrichment analysis of DEGs and DEPs

**FIGURE 2:**
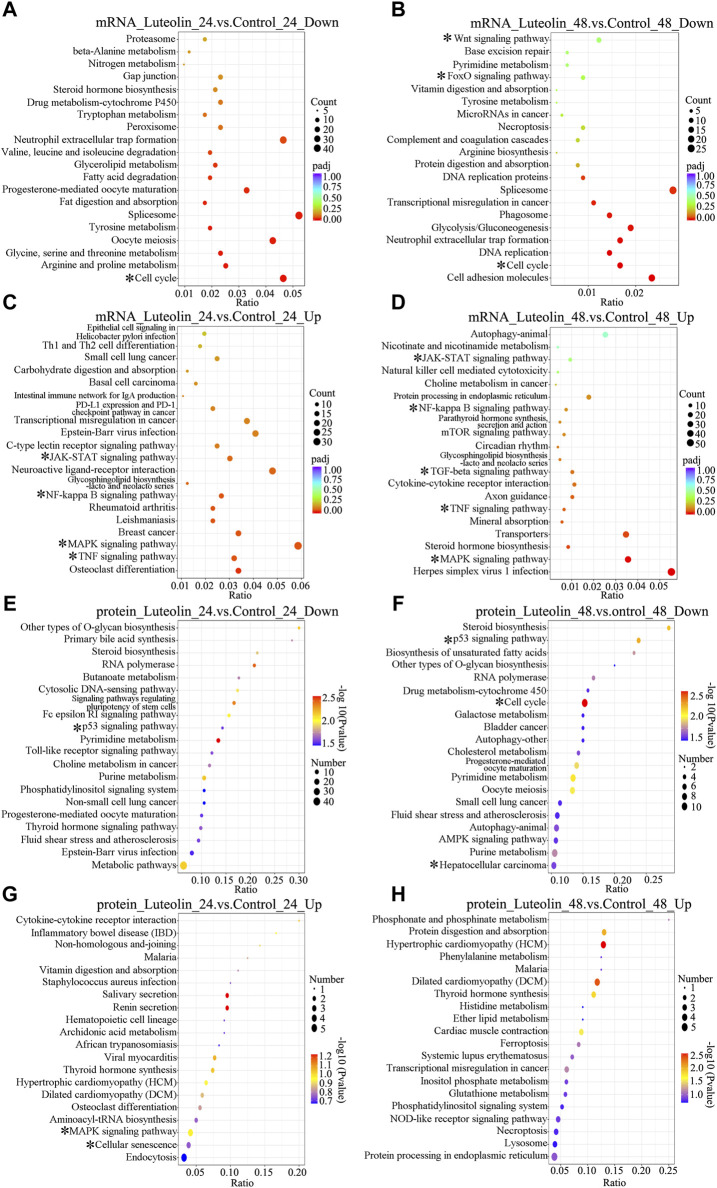
Top 20 significant enriched KEGG pathways of DEGs at the mRNA **(A–D)** level and protein **(E–H)** level of the Luteolin_24 group vs. the Control_24 group **(A,C,E,G)** and the Luteolin_48 group vs. the Control_48 group **(B,D,F,H)**. **(A–D)** The color of the circles is displayed as a gradient from red to purple (mRNA), representing the adjusted *p*-values for each KEGG pathway, with red indicating a more significant *p*-value. The size of the circles corresponds to the number of genes affected in each KEGG pathway **(E,F)** The color of the ovals is displayed as a gradient from purple to red (protein), representing the adjusted *p*-values for each KEGG pathway, with purple indicating a more significant *p*-value. The size of the ovals corresponds to the number of genes affected in each KEGG pathway **(A,B,E,F)** Top 20 significant enriched KEGG pathways of DEGs which are downregulated at the mRNA **(A,B)** level and protein **(E,F)** level **(C,D,G,H)** Top 20 significant enriched KEGG pathways of DEGs which are upregulated at the mRNA **(C,D)** level and protein **(G,H)** level. * represents the most interesting enriched pathways.

Luteolin treatment led to significant changes in gene expression and protein profiles (Supplementary Figures S6A–D; Supplementary Figures S7A–D). According to RNA-seq data, a total of 2,884 and 2,882 DEGs were identified at the mRNA level based on the comparison between the Luteolin_24 and Control_24 groups, and the Luteolin_48 and Control_48 groups, respectively; of these, 1,610 and 1,637 DEGs were upregulated and 1,274 and 1,245 were downregulated, respectively (Supplementary Figure S6C). The top 10 subcategories in biological process (BP), molecular function (MF), and cellular component (CC) are shown in Supplementary Figure S1D. The top 20 KEGG pathways of the upregulated and downregulated DEGs at 24 h and 48 h are shown in Figure 2. Among these pathways, cell cycle, autophagy, MAPK signaling pathway, tumor necrosis factor (TNF) signaling pathway, TGF-β signaling pathway, NF-κB signaling pathway, and NOD-like receptor signaling pathway were related to cell proliferation and cell survival, indicating that luteolin probably inhibited the proliferation of HuH-7 cell via these pathways (Figures 2A–D).

According to proteomics data, a total of 373 and 472 DEGs were identified at the protein level based on comparison between the Luteolin_24 and Control_24 groups, and the Luteolin_48 and Control_48 groups, respectively; of these, 94 and 110 DEGs were upregulated and 279 and 362 were downregulated, respectively (Supplementary Figure S2C). The top 10 subcategories in BP, MF, and CC are shown in Supplementary Figure S2D. The top 20 KEGG pathways of the upregulated and downregulated DEGs at 24 h and 48 h are shown in Figure 2. Among these pathways, the p53 signaling pathway, cell cycle, autophagy, apoptosis, mTOR signaling pathway, TNF signaling pathway, and MAPK signaling pathway were related to cell death, cell proliferation, and cell survival, indicating that luteolin probably inhibited the proliferation of HuH-7 cell via these pathways (Figures 2E–H).

#### 3.2.3 The enriched pathways involved in cell cycle arrest and cell apoptosis

As for the most interesting enriched pathways highlighted with asterisks, were either positively or negatively affected, as shown in Table 1 (24 h) and Table 2 (48 h). The pathways, including cell cycle, JAK-STAT signaling pathway, NF-kappa B signaling pathway, MAPK signaling pathway, Wnt signaling pathway, hepatocellular carcinoma and TGF-beta signaling pathway, promote cell proliferation and interfere with cell apoptosis; while the pathways, including p53 signaling pathway, TNF signaling pathway, Foxo signaling pathway and cellular senescence, inhibit cell proliferation and induce cell apoptosis. To sum up, the transcriptomic and proteomic data showed that luteolin inhibited the proliferation of HuH-7 cells through affecting several signaling pathways relating to cell cycle arrest and cell apoptosis.

**TABLE 1:** The enriched pathways in cells treated with luteolin at 24 h.

Down or Up	Transcriptomic or proteomic	Pathways	Effect	Cell proliferation	Cell apoptosis
Down	Transcriptomic	Cell cycle	Negative	Promote	Interfere
	Proteomic	p53 signaling pathway	Positive	Inhibit	Induce
Up	Transcriptomic	JAK-STAT signaling pathway	Negative	Promote	Interfere
		NF-kappa B signaling pathway	Negative	Promote	Interfere
		MAPK signaling pathway	Negative	Promote	Interfere
		TNF signaling pathway	Positive	Inhibit	Induce
	Proteomic	MAPK signaling pathway	Negative	Promote	Interfere
		Cellular senescence	Positive	Inhibit	Induce

**TABLE 2:** The enriched pathways in cells treated with luteolin at 48 h.

Down or Up	Transcriptomic or proteomic	Pathways	Effect	Cell proliferation	Cell apoptosis
Down	Transcriptomic	Wnt signaling pathway	Negative	Promote	Interfere
		Foxo signaling pathway	Positive	Inhibit	Induce
		Cell cycle	Negative	Promote	Interfere
	Proteomic	p53 signaling pathway	Positive	Inhibit	Induce
		Cell cycle	Negative	Promote	Interfere
		Hepatocellular carcinoma	Negative	Promote	Interfere
Up	Transcriptomic	JAK-STAT signaling pathway	Negative	Promote	Interfere
		NF-kappa B signaling pathway	Negative	Promote	Interfere
		TGF-beta signaling pathway	Negative	Promote	Interfere
		TNF signaling pathway	Positive	Inhibit	Induce
		MAPK signaling pathway	Negative	Promote	Interfere
	Proteomic	-	-	-	-

### 3.3 Possible molecular mechanism revealed by integrating transcriptomics and proteomics data

#### 3.3.1 General insights into the signalling pathways involved in the inhibitory effect of luteolin on hepatocellular carcinoma in HuH-7 cells

Through transcriptome–proteome association analysis, the Venn diagrams of DEGs at the mRNA and protein levels showed a total of 1,152 and 1,071 DEGs, of which 60 and 35 were significantly different (Supplementary Figure S8A). The correlation coefficients between transcriptomic and proteomic expression levels in HuH-7 cells treated with 20 µM luteolin for 24 h and 48 h were 0.049 and 0.121, respectively (Supplementary Figure S8B).

**FIGURE 3:**
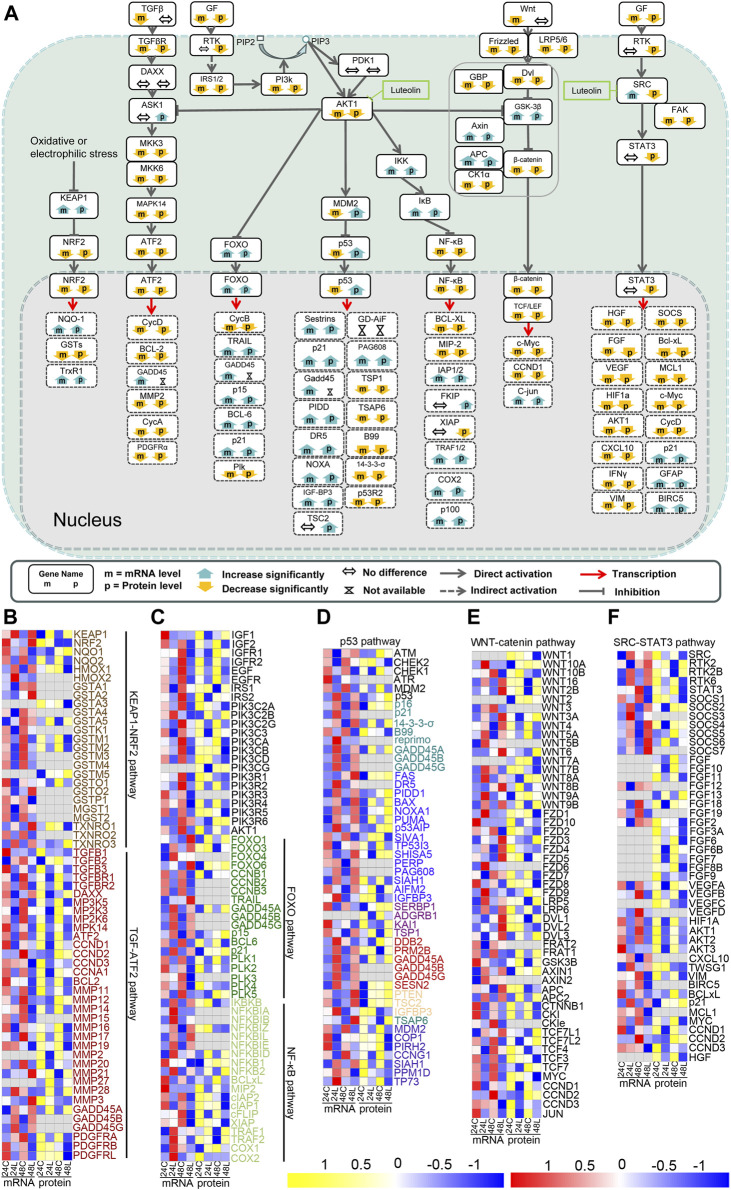
Signaling pathways related to hepatocellular carcinoma are altered in luteolin-treated HuH-7 cells. **(A)** Overview of the signaling pathways related to hepatocellular carcinoma based on the integrated proteogenomic analysis. mRNA and protein abundance of HuH-7 cells treated with luteolin are indicated in comparison with HuH-7 cells without luteolin treatment. Functional categories of representative genes and proteins modulated by luteolin in HuH-7 cells: **(B)** KEAP1-NRF2 pathway and TGF-ATF2 pathway; **(C)** PI3K-AKT-FOXO pathway and PI3K-AKT-NF-κB pathway; **(D)** PI3K-AKT-p53 pathway; **(E)** WNT-catenin pathway; **(F)** SRC-STAT3 pathway. 24C, Control_24; 24L, Luteolin_24; 48C, Control_48; 48L, Luteolin_48. Ratios are color coded as indicated by the color index bar.

For general insights into the signaling pathways involved in the inhibitory effect of luteolin in hepatocellular carcinoma in HuH-7 cells, the transcriptomics and proteomics data across all four groups were integrated and the results are shown in Figures 3A–E. Significant upregulation of key components downstream of the AKT1-MDM2-p53 pathway (p21, GADD45, DR5, etc.) and AKT1-Foxo pathway (TRAIL, p15, p21, etc.) was observed in HuH-7 cells after treatment with luteolin for 24 h and 48 h, indicating enhanced activation of the p53 and Foxo signalling pathways. Considering the inactivation of p53 and Foxo by AKT1, it is probable that the activity of AKT1 was inhibited in HuH-7 cells after treatment with luteolin. Dramatic downregulation of components downstream of the AKT1-ASK2-ATF2 pathway (CycD, BCL2, CycA, etc.), the AKT1-NF-κB pathway (BCL-XL and MIP2) and the AKT1-GSK3β-β-catenin pathway (c-Myc and CCND1) and was observed at the transcriptomic and proteomic levels, indicating increased inactivation of MAPK, NF-κB, β-catenin and JAK-STAT signalling pathways in HuH-7 cells. Considering the activation of ASK2, NF-κB and GSK3β by AKT1, it is also probable that the activity of AKT1 was inhibited in HuH-7 cells after treatment with luteolin. In addition, downregulation of components downstream of the SRC-STAT3 pathway (HGF, AKT1 and CycD, etc.) indicated that SRC was probably inhibited in HuH-7 cells after treatment with luteolin.

#### 3.3.2 Pathways involved in the luteolin-induced cell cycle arrest via AKT1 in HuH-7 cells

**FIGURE 4:**
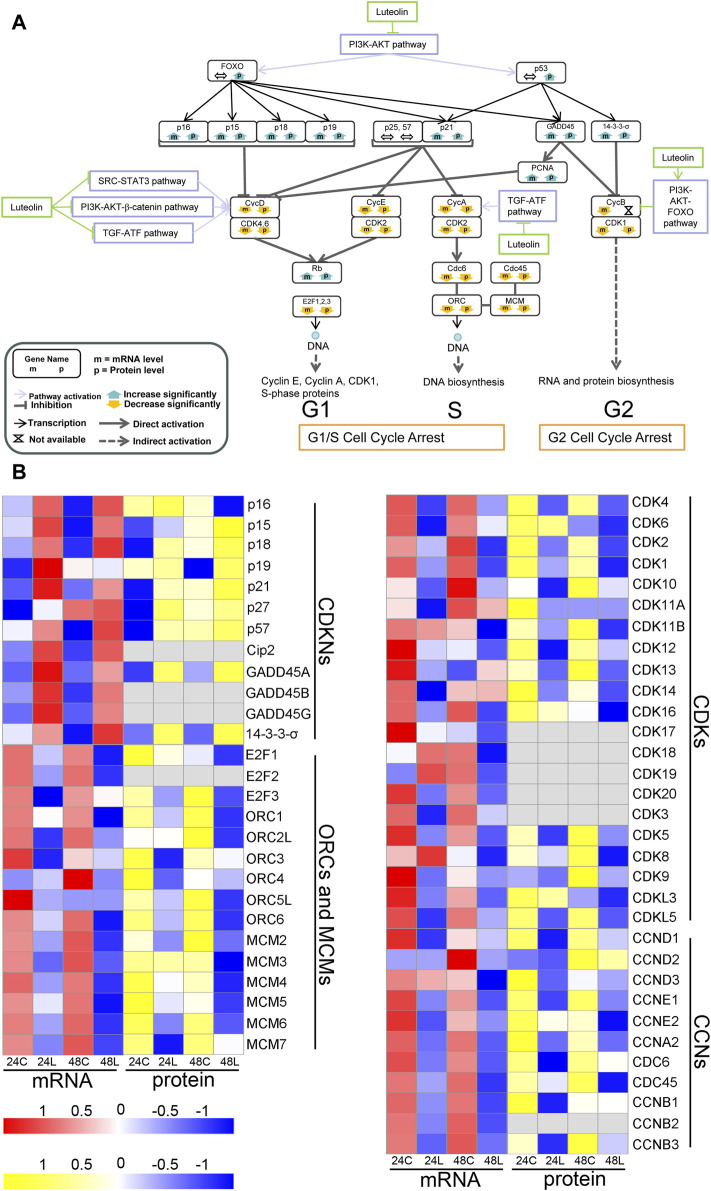
Ideogram illustration of dynamic changes underlying the luteolin-induced cell cycle arrest in HuH-7 cells. **(A)** Overview of genes related to cell cycle arrest based on the integrated proteogenomic analysis. mRNA and protein abundance of luteolin-treated HuH-7 cells are indicated in comparison with HuH-7 cells without luteolin treatment. **(B)** Functional categories of representative genes and proteins modulated by luteolin in HuH-7 cells: CDKNs; ORCs and MCMs; CDKs; CYCs. 24C, Control_24; 24L, Luteolin_24; 48C, Control_48; 48L, Luteolin_48. Ratios are color coded as indicated by the color index bar.

Pathways involved in the luteolin-induced cell cycle arrest in HuH-7 cells are listed in Figure 4. After HuH-7 cells were treated with luteolin for 24 h, p15, p21, GADD45, and 14-3-3-σ were upregulated through the PI3K-AKT1-FOXO/p53 signaling pathway, and the functions of cyclin-dependent kinase (CDK)1, CDK2, CDK4, and CDK6 were consequently inhibited (Figure 4A). Moreover, the expression of cyclin E (CycE), CycA, and CDK1 genes as well as the genes related to S-phase proteins, DNA biosynthesis, and RNA and protein biosynthesis were inhibited (Figure 4A), resulting in cell cycle arrest. The expression of CycD, CycB, and CycA can also be controlled by the SRC-STAT3 pathway, PI3K-AKT-catenin pathway, TGF-ATF, and PI3K-AKT-FOXO pathway (Figure 4A). Changes in the expression of genes involved in cell cycle arrest are shown in Figure 4B.

#### 3.3.3 Pathways involved in the luteolin-induced cell apoptosis via AKT1 in HuH-7 cells

**FIGURE 5:**
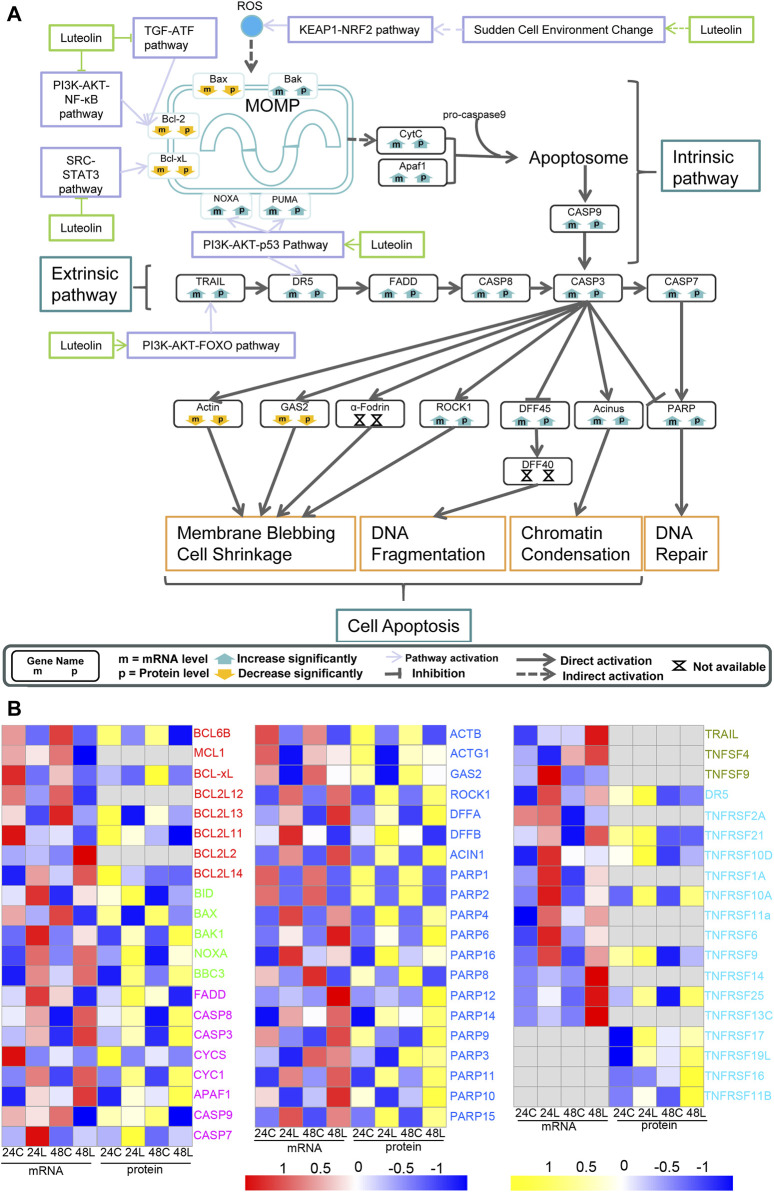
Ideogram illustration of the dynamic changes underlying luteolin-induced apoptosis in HuH-7 cells. **(A)** Overview of genes related to apoptosis based on the integrated proteogenomic analysis. mRNA and protein abundance of luteolin-treated HuH-7 cells are indicated in comparison with HuH-7 cells without luteolin treatment. **(B)** Functional categories of representative genes and proteins modulated by luteolin in HuH-7 cells: BCLs in red; BIDs in green; CASPs in purple; various enzymes in blue; TNFs in dark green; TNFRs in bright blue. 24C, Control_24; 24L, Luteolin_24; 48C, Control_48; 48L, Luteolin_48. Ratios are color coded as indicated by the color index bar.

The expression change of NQO-1, GSTs, and TRXR1 indicated the increase in ROS (Figure 3B in orange). The activated intrinsic mitochondrial pathway of cell apoptosis exercised cell apoptosis together with the extrinsic apoptosis pathway activated by upregulated TRAIL and DR5 through the PI3K-AKT1-FOXO signaling pathway and the PI3K-AKT1-p53 signaling pathway, respectively, eventually leading to HuH-7 cell death (Figure 5). Additionally, the key components in apoptosis, such as BCL-2 and BCL-XL, were downregulated via the PI3K-AKT1-IKBKB-NF-κB signaling pathway and the SRC-STAT3 signaling pathway (Figures 3A, 5A). NOXA was upregulated via the PI3K-AKT1-p53 pathway (Figure 5A).

#### 3.3.4 Pathways involved in the luteolin-induced cell cycle arrest and cell apoptosis via SRC in HuH-7 cells

HGF, FGF, vascular endothelial growth factor (VEGF), HIF1a, Akt1, CXCL10, IFNγ, SOCS, BCL-XL, MCL1, c-Myc, and CycD were downregulated, and p21, GFAP, and BIRC5 were upregulated via the SRC-STAT3 signaling pathway (Figure 3F) and were mainly related to cell cycle arrest and the intrinsic apoptosis pathway (Figures 4A, 5A). Analysis by integrating transcriptomics and proteomics data showed that luteolin inhibited cell proliferation in HuH-7 cells through multiple signalling pathways related to AKT1 or SRC.

### 3.4 Luteolin induced cell cycle arrest and apoptosis in HuH-7 cells and combined directly with AKT1 and SRC

**FIGURE 6:**
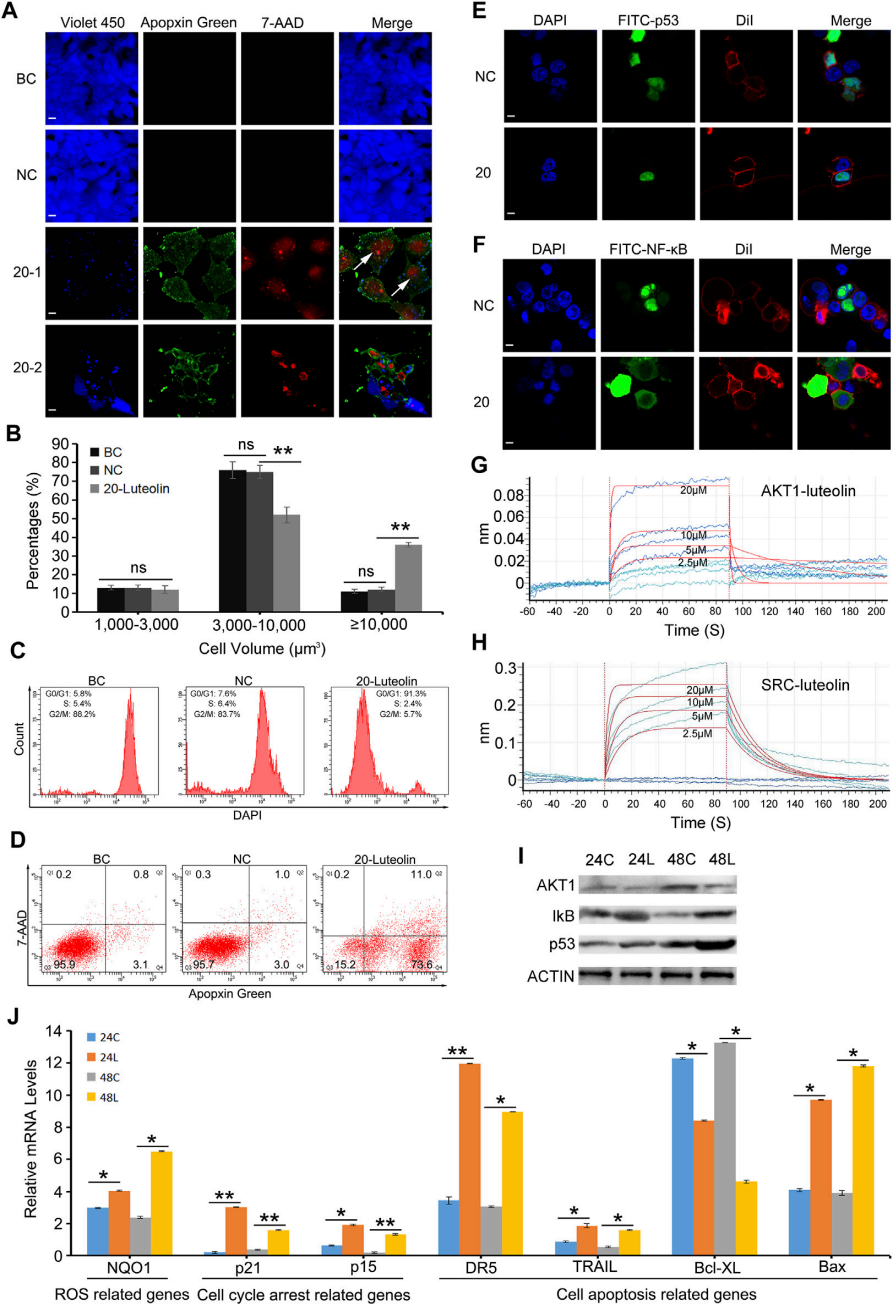
Luteolin inhibits HuH-7 cell proliferation and promotes cell apoptosis via the Akt1-p53/NF-κB signaling pathway. **(A)** Representative CLSM images of apoptotic HuH-7 cells treated with 20 μM luteolin for 24 h. CytoCalcein Violet 450 (blue color, representing live cells), Apopxin Green (green color), 7-AAD (red color), and all three fluorescence stains (merge). BC, blank control; NC, negative control; 20–1, a representative CLSM image of HuH-7 cells treated with 20 μM luteolin for 24 h; 20–2, another representative CLSM image of HuH-7 cells treated with 20 μM luteolin for 24 h. White arrows represent cells that are extremely enlarged in volume. Bars represent 10 μm. Data are representative of four independent experiments. **(B)** Percentages of cells with different volumes. Data are expressed as the mean ± STDEV (n = 3). ns, not significant; **, P < 0.01. **(C)** Representative FACS plots of cells treated with 20 μM luteolin for 24 h stained with DAPI to identify cell cycle stage. Data are representative of four independent experiments. **(D)** Representative FACS plots of cells treated with 20 μM luteolin for 24 h and stained with Apopxin Green and 7-AAD to identify apoptotic cells in the early and late stages, respectively. Data are representative of four independent experiments **(E,F)** Representative CLSM images of the translocation of the transcription factor p53 **(E)** and NF-κB **(F)**. DAPI (blue color, representing cell nucleus), FITC (green color, representing the location of the transcription factor p53 **(E)** or NF-κB **(F)**, DiI (red color, representing the cell membrane), and merge (representing all three fluorescence stains). Bars represent 10 μm. Data are representative of four independent experiments **(G,H)** BLI binding results of different concentrations of luteolin with AKT1 **(G)** and SRC **(H)**. Data are representative of four independent experiments. **(I)** Protein levels of AKT1 and the related downstream genes of AKT1 and SRC were detected by Western blot, and actin was used as a control **(J)** Several key genes’ mRNA expression level by qRT-PCR assay. Data are expressed as the mean ± STDEV (n = 4). *, P < 0.05; **, P < 0.01.

CLSM and flow cytometry were used to analyse cell cycle arrest and apoptosis, which are predicted to be involved in the proliferation of liver cancer cells were analyzed. CLSM analysis showed that the cell cycle arrest occurred in HuH-7 cells after treatment with luteolin for 24 h. The increased cell volume was observed in the 20 µM-luteolin treated group (Figure 6A–20–1). In addition, we found that the cell volume of all three groups ranged from 1,411 μm^3^ to 58,512 μm^3^ as calculated by Cellsens software. In the BC group, the percentages of cells with volumes of 1,000–3,000 μm^3^ were (13.33 ± 1.31)%, 3,000–10,000 μm^3^ were (76.15 ± 4.50)%, and >10,000 μm^3^ were (11.35 ± 1.14)%; In the NC group, the percentages were (12.54 ± 1.43)% (75.23 ± 3.57)% and (12.16 ± 1.38)%, respectively; In the 20 µM-luteolin-treated group, the percentages were (12.97 ± 2.15)% (52.30 ± 4.21)% and (36.48 ± 1.21)%, respectively (Figure 6B). Furthermore, the cell cycle was analyzed by flow cytometry, and the results showed that the percentages of cells of the BC group in G0/G1 were (5.8 ± 1.16)%, in S were (5.4 ± 0.86)%, and in G2/M were (88.2 ± 3.65)%, respectively; of the NC group were (7.6 ± 1.77)% (6.4 ± 2.33)% and (83.7 ± 2.97)%, respectively; of the 20 µM-luteolin treated group were (91.3 ± 3.36)% (2.4 ± 0.79)% and (5.7 ± 2.88)%, respectively (Figure 6C). As for cell apoptosis, CLSM analysis showed that apoptotic cells of 20 µM-luteolin-treated HuH-7 cells increased significantly, as the Violet 450 staining became weaker and the Apopxin Green and 7-AAD staining became stronger of the 20 µM-luteolin-treated HuH-7 cells when compared with either the HuH-7 cells of the BC group or the HuH-7 cells of the NC group (Figure 6A–20–2). In parallel, flow cytometry assay results showed that the cell apoptosis percentage (Q2+Q4) of the three groups were (3.9 ± 1.85)% (BC group) (4.0 ± 0.71)% (NC group) and (84.6 ± 2.97)% (20 µM-luteolin-treated group) (Figure 6D). In addition, CLSM analysis revealed more nuclear p53 cells and fewer nuclear NF-κB cells in HuH-7 cells compared to the negative control by immunofluorescence staining assay (Figures 6E, F). Futhermore, the combination of luteolin with AKT1 and SRC was further tested using BLI, and the combination affinity KD (Mol) between them was determined to be 7.857 × 10^−6^ and 1.700 × 10^−6^ (Figures 6G, H). Taken together, these results favored the hypothesis that luteolin directly combined with AKT1 and SRC, inhibiting their activity, and ultimately leading to cell death in HuH-7 cells. The protein levels of AKT1 and the related downstream genes of AKT1 and SRC were verified to be reliable by Western blotting (Figure 6I). Using qRT-PCR, the expression of several key genes related to ROS, cell cycle arrest and cell apoptosis was verified to be reliable (Figure 6J).

## 4 Discussion

### 4.1 The inhibition effect of luteolin on HuH-7 cells

As a predictor of the clinical outcome of the drug in clinical trials, the assessment of the safety and efficacy of a drug candidate is a critical part of drug development (Muller and Milton, 2012). Luteolin has been developed as a health food for commercial use and has also been included in cosmetic products owing to its safety profile and various biological effects. Luteolin is nontoxic; the oral median lethal dose (LD_50_) was found to be >2,500 and 5,000 mg/kg in mice and rats, respectively, which is equivalent to approximately 219.8–793.7 mg/kg in humans (Aziz et al., 2018). In our study, the inhibitory effect of luteolin on the proliferation of HuH-7 liver cancer cells was determined to be time- and dose-dependent (Figure 1). A similar inhibitory effect of luteolin on hepatocarcinoma cells has been reported on the basis of *in vitro* studies using SMMC-7721, BEL-7402, and HepG2 hepatocarcinoma cells (Hwang et al., 2011; Yee et al., 2015) and *in vivo* studies using xenograft tumor mice (Hwang et al., 2011). In addition, a previous study showed that luteolin reduced the viability of three subtypes of NSCLC cells, including the T790 M mutant NSCLC cells, and did not affect the viability of L02 (a normal liver cell line), H9c2 (a normal cardiomyocyte cell line) and HEK293 (a normal kidney cell line) cell lines (Hong et al., 2014). The therapeutic efficacy of a drug is also highly dependent on its bioavailability (Khan and Singh, 2016). The low bioavailability of dietary flavonoids limits their biological effects *in vivo*, which has always been a major problem for their pharmaceutical applications (Gao and Hu, 2010). For luteolin, the oral bioavailability in rats is only 26% ± 6% (Lin et al., 2015). However, after oral administration, luteolin showed relatively rapid absorption and slow elimination in rats, with a t_max_ (time to reach peak plasma level) of approximately 1.02 h and a t_1/2_ (elimination half-life) of 4.94 h, indicating that luteolin may accumulate in the plasma after repeated administration (Zhou et al., 2008). In summary, these findings indicate the potential of luteolin for development as a safe and effective therapeutic agent to treat liver cancer.

### 4.2 The potential pathways involved with cell cycle arrest and apoptosis

**FIGURE 7:**
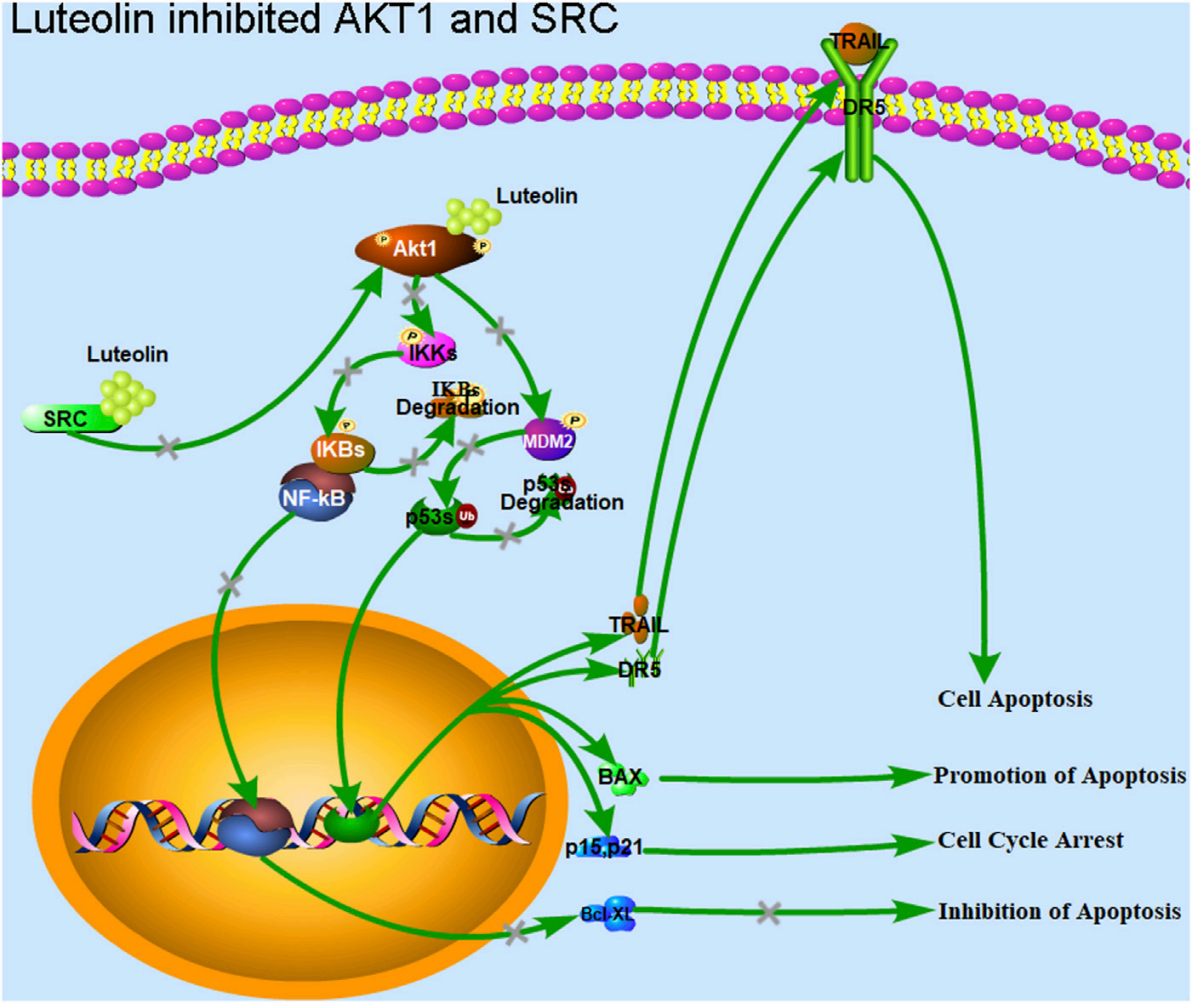
Luteolin inhibits the proliferation of liver cancer cells via targeting AKT1 and SRC. Luteolin promotes cell cycle arrest and apoptosis in HuH-7 cells through the transcription factors p53 and NF-κB via AKT1 and SRC. AKT1 is involved in the MDM2-p53 and IKK-NF-κB pathways, and regulates the expression of the genes related to ROS, cyclin, BCL2s, and CDKNs. AKT1 inhibition by luteolin promotes the function of the transcription factor p53, and inhibits the function of the transcription factor NF-κB. The expression of genes such as BCL2s associated with apoptosis inhibition, and those associated with cell cycle progression, including cyclins and CDKs, were inhibited. Genes associated with promoting apoptosis, such as TRAIL and DR5, and those associated with cell cycle arrest, such as CDKNs and GADD45, were upregulated. Finally, cell cycle arrest, apoptosis, and cell death were achieved. The inhibition of SRC led to a downregulation of AKT1 expression, which has the same effect as the direct inhibition of AKT1 function.

By integrating transcriptomic and proteomic data and based on KEGG analysis, we found that significant changes in the expression of genes downstream of the KEAP1-NRF2, PI3K-AKT1-ASK1-ATF2, PI3K-AKT1-FOXO, PI3K-AKT1-p53, PI3K-AKT1-IKBKB-NF-κB, PI3K-AKT1-GSK3β-β-catenin-TCF/LEF, and SRC-STAT3 signaling pathways were observed in the Luteolin_24 and Luteolin_48 groups, indicating that these pathways were activated in HuH-7 cells after luteolin treatment (Figure 3; Figure 7). Most pathways mentioned above overlap with the pathways predicted using network pharmacology (Mo et al., 2023) and have been well documented (Krause et al., 2000; Vousden and Prives, 2009; Böhlig and Rother, 2011). However, the expression levels of some molecules may be altered due to indirect effects of luteolin, rather than being directly involved in luteolin-induced apoptotic pathways. To strengthen this hypothesis, we would prefer to conduct experiments such as knockout/knockdown of AKT1/SRC or their downstream target genes, followed by treatment with luteolin to observe their effects on cell cycle arrest and cell apoptosis dynamics in the future study, thus validating the proposed pathways in Figure 7 to distinguish between association and causation for each pathway in the anticancer effects of luteolin.

### 4.3 AKT1 is the direct target of luteolin

In our study, we found that HuH-7 cells underwent apoptosis after luteolin treatment (Figures 6A, B). Furthermore, cell volume enlargement indicated that HuH-7 cells also underwent cell cycle arrest (Figure 6A). AKT1 participates directly or indirectly in most of these pathways relating to apoptosis and cell cycle arrest (Nitulescu et al., 2016), i.e., phosphorylating multiple Ser/Thr residues of its downstream targets, FOXO3A, MDM2, GSK3β, ASK1, TSC2, IKK, and WEE1, and producing a long-term effect in reducing apoptosis and promoting cell survival (Cantley, 2002; Wagener et al., 2017). In our study, BLI assay revealed that luteolin could combine with active AKT1 in a dose-dependent manner at the molecular level with a KD at 10^–6^ M (Figure 6E). Coincidentally, molecular docking analysis has also shown that luteolin can directly interact with AKT1 with relatively low energy (Mo et al., 2023), and three amino acid residues-namely Tyr^175^, Asn^231^, Glu^432^-in AKT1 have been modelled to form hydrogen bonds with luteolin (Hu et al., 2024). In addition, the mammalian AKT1 isoforms are encoded by different genes and share the structural feature of three functional domains: an N-terminal PH (pleckstrin homology) domain (Ala^5^-Asp^108^) which is essential for binding to lipids such as PtdIns(3,4,5) P3, a central catalytic domain (Phe^150^-Phe^408^) related to protein kinases A and C, and a C-terminal regulatory tail (Phe^408^-Ala^480^) (Hanada et al., 2004). It seems that it is the catalytic domain and the regulatory tail of AKT1 that luteolin binds to. In future studies, point mutation experiments can be designed to confirm this conclusion and provide further insight into the mechanism of action. These results suggest AKT1 to be the likely direct target of luteolin in HuH-7 cells and mediate the cell cycle arrest and apoptosis (Figure 7).

AKT1 can inactivate p53, which can coordinate multiple responses including cell cycle arrest, DNA repair, metabolic changes, antioxidant effects, anti-angiogenic effects, autophagy, aging, and apoptosis (Bieging et al., 2014), by phosphorylating its ubiquitin ligase MDM2, thereby inhibiting p53-induced cell cycle arrest and apoptosis (Ogawara et al., 2002; Vassilev et al., 2004). The significant nuclear translocation increasing of p53 (Figure 6C), the upregulation of p21, GADD45, PAG608, DR5, PIDD, IGFBP3, PTEN, 14-3-3-σ, and NOXA, and the downregulation of B99 and p53R2 at the mRNA and protein levels (Figure 3A) implied that luteolin probably induced cell cycle arrest and cell apoptosis by inhibiting AKT1 activity, thereby inhibiting MDM2 activity and promoting p53 activity in HuH-7 cells (Figures 3–5).

Except for p53, AKT1 also regulates the transcription factor NF-κB positively by regulating IKK, thereby promoting cell survival, proliferation, invasion, and angiogenesis, and resistance to chemotherapy (Zhang et al., 2005). Moreover, it negatively regulates ATF2 by regulating ASK1, thereby inhibiting cell apoptosis (Wagener et al., 2017), eventually regulating the expression of BCL-XL and BCL2. In this study, BCL2, BCL2L1, BCL2L11, BCL2L13, and BCL2L14 were downregulated and Bax was unchanged after HuH-7 cells were treated with luteolin (Figure 5B). The downregulation was likely attributable to NF-κB and ATF, which increased the Bax/Bcl-2 ratio. The synergistic antitumor effect of luteolin and 5-fluorouracil is related to the increased Bax/Bcl-2 ratio in human hepatocellular carcinoma cells (Xu et al., 2016). As early as 1993, Korsmeyer et al. (1993) proposed that the Bcl-2/Bax ratio is the “variable resistor” (rheostat) that regulates cell death. Their hypothesis was confirmed subsequently by multiple studies on the Bcl-2/Bax ratio as a clinical prognostic marker of cancer. The significant nuclear translocation decreasing of NF-κB and the downregulation of BCL-XL and BCL-2 implied that luteolin probably induced apoptosis by inhibiting AKT1 activity, which, in turn, inhibited NF-κB activity and promoted ATF2 activity in HuH-7 cells (Figures 5A, 6D).

Moreover, luteolin treatment arrested cell cycle progression suddenly via AKT1 by destroying the normal metabolism in HuH-7 cells and resulted in the accumulation of reactive oxygen species (ROS) (Hayes et al., 2020), which was related to MOMP activation, and further activated cell apoptosis (Figure 7). A study has demonstrated that luteolin can induce a lethal endoplasmic reticulum stress response and mitochondrial dysfunction in glioblastoma cells by increasing intracellular reactive oxygen species (ROS) levels (Wang et al., 2017).In addition, luteolin also acts as a radiosensitizer in non-small cell lung cancer cells by enhancing apoptotic cell death through activation of a p38/ROS/caspase cascade (Cho et al., 2015). In fact, ROS plays a dual role in promoting cancer development and killing cancer cells depending on its concentration and the cellular context (Hayes et al., 2020; Lei et al., 2021). It is fundamentally important for cancer cells to achieve a balance between ROS levels and oxidative damage that a cell can bear (Cui et al., 2018; Hayes et al., 2020). The change in expression of the genes NQO-1, GSTs, and TRXR1 indicated disruption of the ROS balance in HuH-7 cells after luteolin administration and the cells attempting to maintain a higher balance of the ROS/antioxidant level (Figures 3A, B). Furthermore, the accumulating ROS in HuH-7 cells had a considerable effect on the function and structure of the mitochondria and promoted MOMP and CytC leaking (Tabatabaie et al., 2022). Next, CASP9 as well as the apoptosome and CASP3 were activated (Tabatabaie et al., 2022). These findings were consistent with the hypothesis that an increase in ROS by drugs is an important therapeutic strategy to overcome multidrug resistance in cancer cells (Cui et al., 2018).

### 4.4 SRC is another target of luteolin

SRC is another probable target protein of luteolin in HuH-7 cells. It has been demonstrated that the proliferation induced by stable LHB expression is associated with increased G1/S cell cycle progression and apoptosis resistance mediated by SRC kinase activation, as established using clinical specimens of hepatocellular carcinoma (Liu et al., 2011). In addition, activated SRC can activate STAT3 and lead to its constitutive activation. Phosphorylated STAT3 dimerizes and translocates to the nucleus, which causes the transcription of target genes including those related to immunosuppression, angiogenesis, metastasis, proliferation, and survival (Zou et al., 2020). Changes in the expression of the genes mentioned above indicated inhibition of the SRC-STAT3 pathway (Figure 3). It has been demonstrated that luteolin exerts antimelanoma effects *in vitro* and *in vivo* without overt toxicity to normal cells and also in melanoma-bearing mice through the suppression of STAT3 signaling via binding to SRC protein (Li et al., 2022). Notably, molecular docking studies have reported that luteolin can directly interact with SRC with rather low energy (Mo et al., 2023). Future studies should focus on the differences in the binding affinity of luteolin with AKT1 and SRC to identify the protein that functions independently in the inhibition of liver cancer when using luteolin to further enhance its use.

In conclusion, luteolin significantly inhibited HuH-7 cell proliferation in a dose- and time-dependent manner. The combined transcriptomic and proteomic approach revealed that luteolin could promote cell cycle arrest and apoptosis through the transcription factors p53 and NF-κB via direct binding to AKT1 in HuH-7 cells, as well as through the SRC-STAT3 pathways via direct binding to SRC (Figure 7). Our results systematically elucidate the mechanism of luteolin in inhibiting liver cancer cells, mainly through cell cycle arrest and apoptosis via targeting AKT1 and SRC. Further clinical studies are required to determine the accurate dose and safety of luteolin in treating liver cancer. Moreover, modifying structure of luteolin to improve its ability to bind to target molecules is an aspect that should be pursued.

## Figures and Tables

**Table:** 

**HuH-7**	human hepatoma 7
**CLSM**	confocal laser scanning microscope
**BLI**	bio-layer interferometry
**NF-κB**	nuclear factor kappa B
**FOXO**	forkhead box protein O
**ATF2**	activating transcription factor 2
**TCF/LEF**	T cell factor/lymphocyte enhance factor
**AKT1**	Protein Kinase B α PKBα
**KEAP1**	Kelch like associated protein 1
**NRF2**	nuclear factor erythroid 2-related factor 2
**SRC**	sarcoma
**STAT3**	signal transducer and activator of transcription three
**p21/WAF1/CIP1**	protein 21/wild type p53 activated fragment 1/cyclin-dependent kinase inhibitor 1
**Smad4**	*drosophila* mothers against decapentaplegic protein4
**TGF-β1**	tansforming growth factor-β1
**p27/KIP1**	protein 27/kinase-interacting protein 1
**Fas**	factor related apoptosis
**G1/S phase**	gap1/synthesis phase
**BCL-2**	B-cell lymphoma-2
**BAX**	BCL-2 associated X
**MAP2K1**	mitogen activated protein kinase kinase 1
**EGFR**	epithelial growth factor receptor
**PIK3CA**	phosphatidylinositol-4,5-bisphosphate 3-kinase catalytic subunit α
**MET**	cellular-mesenchymal epithelial transition factor
**MAPK3**	mitogen activated protein kinase 3
**MAP3K7**	mitogen activated protein kinase kinase kinase 7
**PBS**	phosphate-buffered saline
**log2FC**	log2 fold change
**FDR**	false discovery rate
**FITC**	fluoresceine isothiocyanate
**DAPI**	4′,6-diamidino-2-phenylindole
**DiI**	dialkylcarbocyanines I
**DMSO**	dimethyl sulfoxide
**LD50**	median lethal dose
**IC50**	half-maximal inhibitory concentration
**RNA-seq**	RNA sequencing
**DEGs**	differentially expressed genes
**GO**	gene ontology
**BP**	biological process
**MF**	molecular function
**CC**	cellular component
**KEGG**	Kyoto Encyclopedia of Genes and Genomes
**TNF**	tumor necrosis factor
**NLRs**	nucleotide oligomerization domain (NOD)-like receptors
**mTOR**	mechanistic target of rapamycin
**ASK1**	apoptosis signal regulating kinase 1
**IKBKB**	inhibitor of kappa light polypeptide gene enhancer in B-cells, kinase beta
**GSK3β**	glycogen synthase kinase3β
**TSC2**	tuberous plex-2
**KD**	constant of dissociation
**CDKN2B/p15/INK4b**	cyclin dependent kinase inhibitor/protein 15/inhibitor of cyclin dependent kinase 4b
**GADD45**	growth arrest and DNA damage-inducible genes
**CDK**	cyclin-dependent kinase
**CycD**	cyclin D
**CycB**	cyclin B
**CycA**	cyclin A
**CDC**	cell division cycle
**MCM**	minichromosome maintenance proteins
**ORC**	origin recognition complex
**ROS**	reactive oxygen species
**MOMP**	mitochondria outer membrane permeabilization
**NQO-1**	NADH dehydrogenase, quinone 1
**GSTs**	glutathione S-transferases
**TRXR1**	thioredoxin reductase 1
**CytC**	cytochrome C
**CASPs**	caspases
**CASP9**	caspase 9
**CASP3**	caspase 3
**TNFSF**	tumor necrosis factor super family
**TNFRSF**	tumor necrosis factor receptor super family
**TRAIL**	TNF-related apoptosis-inducing ligand
**DR5**	death receptor 5
**BCL-XL**	B-cell lymphoma XL
**BH3**	Bcl-2-homology 3
**BIDs**	BH3 interacting domain death agonist
**BAK**	BCL-2 homologous antagonist/killer
**VEGF**	vascular endothelial growth factor
**HIF1**	hypoxia inducible factor-1
**CXCL10**	C-X-C chemokine ligand-10
**IFNγ**	interferon γ
**SOCS**	suppressors of cytokine signaling
**MCL1**	myeloid cell leukemin-1
**c-Myc**	cellular-myelocytomatosis viral oncogene
**GFAP**	glial fibrillary acidic protein
**IAP**	inhibitor of apoptosis
**BIRC5**	baculoviral IAP repeat-containing 5
**LHB**	luteinizing hormone beta
**7-AAD**	7-amino-actinomycin D
**SEM**	standard error of mean
**CYC**	cytochrome C
**BC**	blank control
**NC**	negative control
**24C**	Control_24
**24L**	Luteolin_24
**48C**	Control_48
**48L**	Luteolin_48
**FACS**	fluorescence activated cell sorting

## Data Availability

The datasets presented in this study can be found in online repositories. The names of the repository/repositories and accession number(s) can be found in the article/Supplementary Material.
