# Supplementary material for: Combination of transcriptomic and proteomic approaches helps unravel the mechanisms of luteolin in inducing liver cancer cell death via targeting AKT1 and SRC
Source: Front Pharmacol. 2024 Aug 21;15:1450847. doi: 10.3389/fphar.2024.1450847 (PMC11371790; doi:10.3389/fphar.2024.1450847)
Supplement: Supplementary file 1 [file DataSheet1.docx]

Supplementary Material

**1 Supplementary Tables and Figures**

- 1. **Supplementary Tables**

**Supplemental Table 1** Summary of sample sequencing data quality

| sample | raw_reads | raw_bases | clean_reads | clean_bases | error_rate | Q20 | Q30 | GC_pct |
| --- | --- | --- | --- | --- | --- | --- | --- | --- |
| control_24_1 | 50199010 | 7.53G | 46953748 | 7.04G | 0.03 | 97.05 | 92.29 | 49.55 |
| control_24_2 | 48961768 | 7.34G | 45868264 | 6.88G | 0.03 | 96.78 | 91.65 | 50.04 |
| control_24_3 | 39403170 | 5.91G | 38482794 | 5.77G | 0.03 | 97.28 | 92.53 | 50.37 |
| luteolin_24_1 | 47313518 | 7.1G | 44739560 | 6.71G | 0.03 | 96.95 | 92.04 | 50.03 |
| luteolin_24_2 | 43652124 | 6.55G | 40832466 | 6.12G | 0.03 | 97.18 | 92.53 | 49.97 |
| luteolin_24_3 | 43126830 | 6.47G | 40756704 | 6.11G | 0.03 | 97.15 | 92.43 | 50.12 |
| control_48_1 | 43595692 | 6.54G | 41301086 | 6.2G | 0.03 | 96.88 | 91.91 | 50.39 |
| control_48_2 | 46584938 | 6.99G | 43539742 | 6.53G | 0.03 | 97.19 | 92.57 | 50.02 |
| control_48_3 | 48072174 | 7.21G | 45052114 | 6.76G | 0.03 | 97.19 | 92.53 | 50.14 |
| luteolin_48_1 | 47546166 | 7.13G | 44987424 | 6.75G | 0.03 | 97.08 | 92.24 | 49.03 |
| luteolin_48_2 | 43981490 | 6.6G | 41491768 | 6.22G | 0.03 | 96.95 | 92 | 49.63 |
| luteolin_48_3 | 50800166 | 7.62G | 49311564 | 7.4G | 0.03 | 97.24 | 92.42 | 49.79 |

**Supplemental Table 2** Signaling pathways and mechanisms involved in luteolin-induced apoptosis and cell cycle arrest in liver cancer cells

| Cells | Signal pathway | Mechanism | Note |
| --- | --- | --- | --- |
| HepG2 cells | Fas/FasL | Induced the FasL-induced apoptosis through inhibiting Akt activation and promoting proteasomal degradation of X-linked Inhibitor of Apoptosis Protein (XIAP) | Liu et al., 2011 |
|  | p53 | Increased the bax/bcl-2 ratios and p53 expressions, enhanced PARP | Xu et al., 2016 |
|  | STAT3/Fas | Caused a large decrease in the Tyr (705) phosphorylation of STAT3, a known negative regulator of Fas/CD95 transcription, which occurred within 20 min in the luteolin-treated cells with an increased expression of Fas/CD95. | Yee et al., 2015 |
|  | AMPK-NF-κB | Caused the release of reactive oxygen species (ROS) and that these intracellular ROS in turn mediate AMPK-NF-κB signaling in HepG2 hepatocarcinoma cells. | Hwang et al., 2011 |
|  | CYP1A-catalyzed metabolism | Induction of G2/M arrest was accompanied by up-regulation of phosphor-extracellular-signal-regulated kinase (p-ERK), phosphor-c-jun N-terminal kinase, p53 and p21 proteins. | Androutsopoulos et al., 2013 |
| Bel7402 cells |  | Increased the bax/bcl-2 ratios and p53 expressions, enhanced PARP | Xu et al., 2016 |
| Mouse H22 hepatoma cells | STAT3 | Up-regulating the expression of ICAM-1, down-regulating the LFA-3 expression, and decreasing the PCNA expression; down-regulation of the target gene products of STAT3 such as surviving Bcl-xL, cyclin D1, and vascular endothelial growth factor. | Niu et al., 2015 |
| liver cancer cell line SMMC-7721, BEL-7402 | Bcl-2 | Enhancing Bax level, reducing anti-apoptotic protein Bcl-2 level, resulting in activating caspase-3 enzyme and decrease of mitochondrial membrane potential, and finally leading to cell apoptosis. | Ding et al., 2014 |
| HLF hepatoma cells | STAT3/Fas | Targeted STAT3 through dual pathways—the ubiquitin-dependent degradation in Tyr705-phosphorylated STAT3 and the gradual down-regulation in Ser727-phosphorylated STAT3 through inactivation of CDK5, thereby triggering apoptosis via up-regulation in Fas/CD95. | Selvendiran et al., 2006 |
| Human hepatoma Huh-7 cells | ROS production | Mediated through effects involving intracellular ROS. | Yoo et al., 2009 |

**Supplemental Table 3** Primer sequences for qRT-PCR analysis

| Primer Name | Primer Sequence (5' to 3') |
| --- | --- |
| NQO1-F | GTCGGCAGAAGAGCACTGATCG |
| NQO1-R | ACTCCACCACCTCCCATCCTTTC |
| p21-F | GTACCCTTGTGCCTCGCTCAG |
| p21-R | TGGTCTGCCGCCGTTTTCG |
| p15-F | ACCGTTCATGTAGCAGCAACCG |
| p15-R | ACCTTGGGACCAGTGTACCTTCTC |
| DR5-F | AGTCACAGTTGCAGCCGTAGTC |
| DR5-R | CCACCACCACCTGAGCAGATG |
| TRAIL-F | GACTCCTGCCTCTCCCTGTTCTC |
| TRAIL-R | ACACAGCCACAATCAAGACTACGG |
| Bcl-XL-F | GCAGCCGAGAGCCGAAAGG |
| Bcl-XL-R | GAAGAGTGAGCCCAGCAGAACC |
| Bax-F | ACCAAGAAGCTGAGCGAGTGTC |
| Bax-R | TGTCCACGGCGGCAATCATC |
| GAPDH-F | CCCCTTCATTGACCTCAACTACA |
| GAPDH-R | AAGACACCAGTAGACTCCACGACAT |

## Supplementary Figures


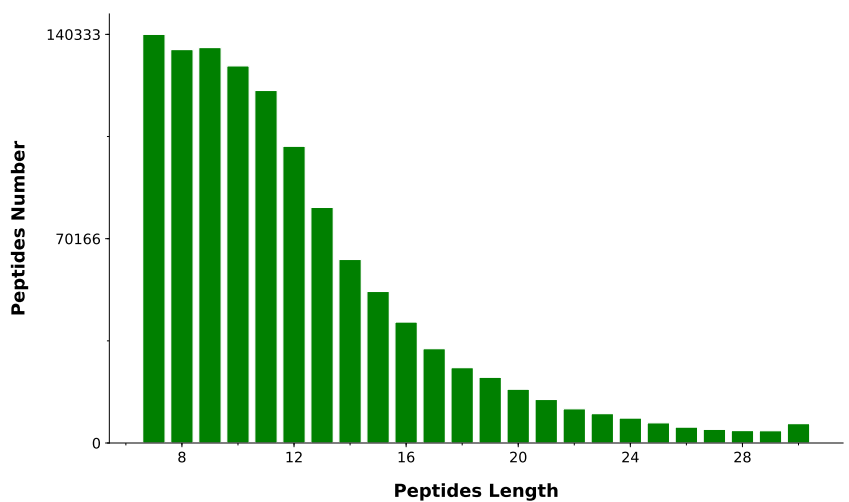


**Supplemental Figure 1. Peptide length range distribution.** The abscissa is the number of amino acid residues of the peptide, and the ordinate is the number of peptides of that length. For routine complex samples, peptide lengths are mainly distributed between 7-25.

**
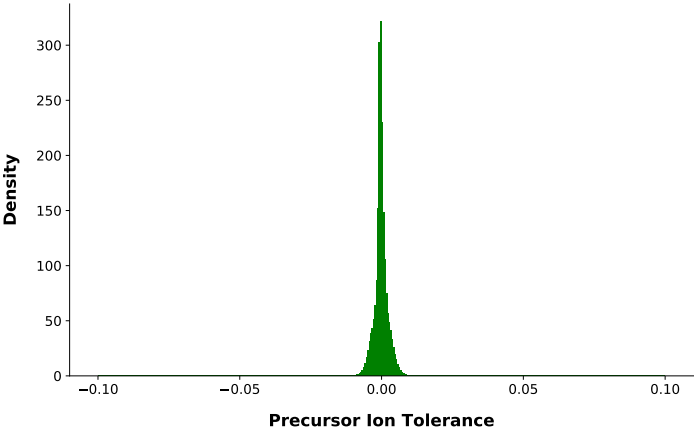
Supplemental Figure 2. Precursor Ion Mass Tolerance Distribution.** The abscissa is the mass deviation, and the ordinate is the precursor ion density distribution of the corresponding error. The more concentrated the peak shape is around 0, the smaller the mass deviation.


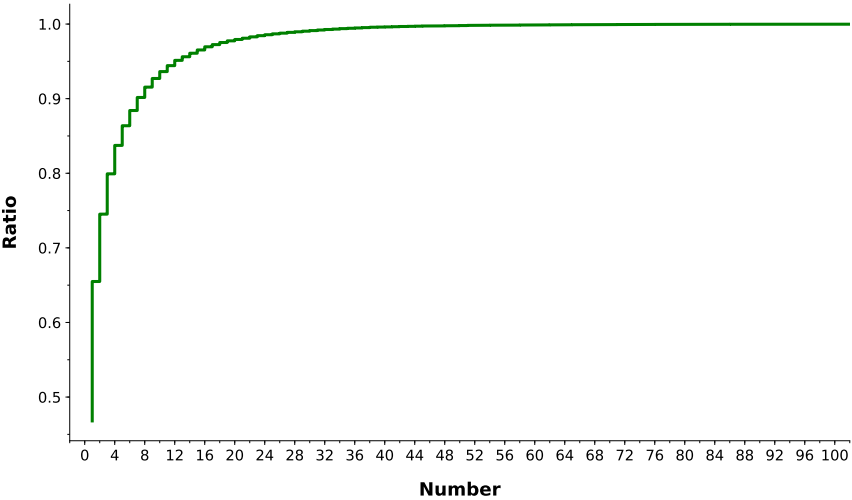


**Supplemental Figure 3. Map of the number of unique peptides in the identified protein.** The abscissa is the number of unique peptides, and the ordinate is the cumulative proportion of proteins containing unique peptides to the total protein as the number of unique peptides increases. Therefore, the slower the increase in the curve, the greater the number of unique peptides, the more reliable proteins were identified.


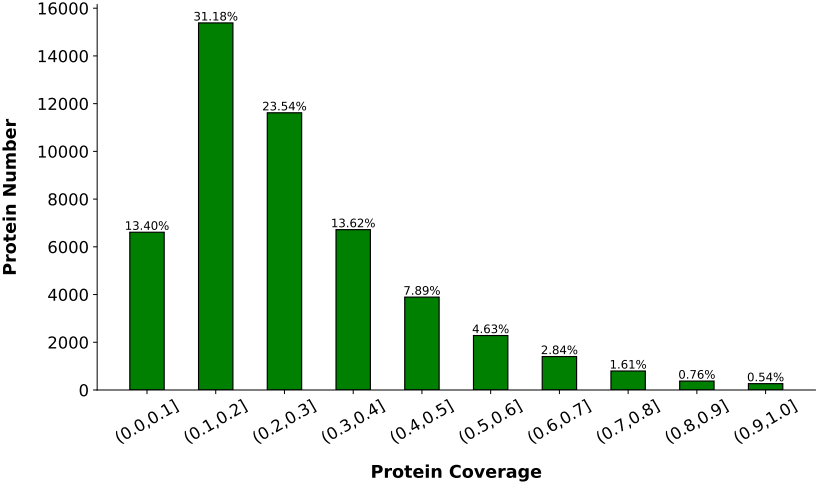


**Supplemental Figure 4. Protein coverage distribution.** The abscissa is the interval of protein coverage (the length of the protein covered by the detected peptide/the full length of the protein), and the ordinate is the number of proteins contained in the corresponding interval.


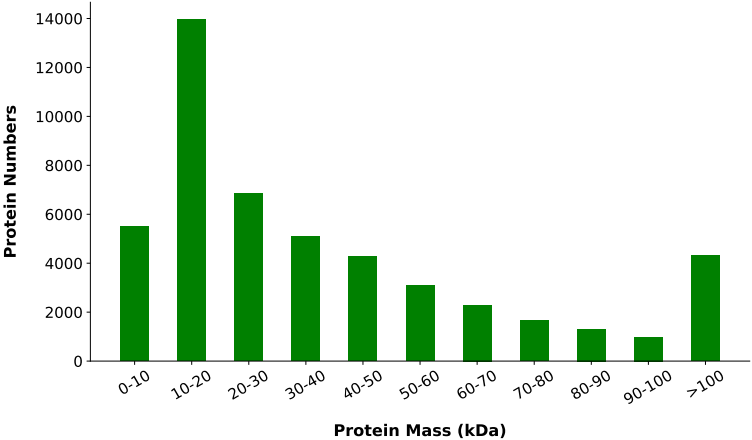


**Supplemental Figure 5. Protein molecular weight distribution.** The abscissa is the molecular mass of the identified protein (unit: thousand daltons, kDa), and the ordinate is the number of identified proteins.


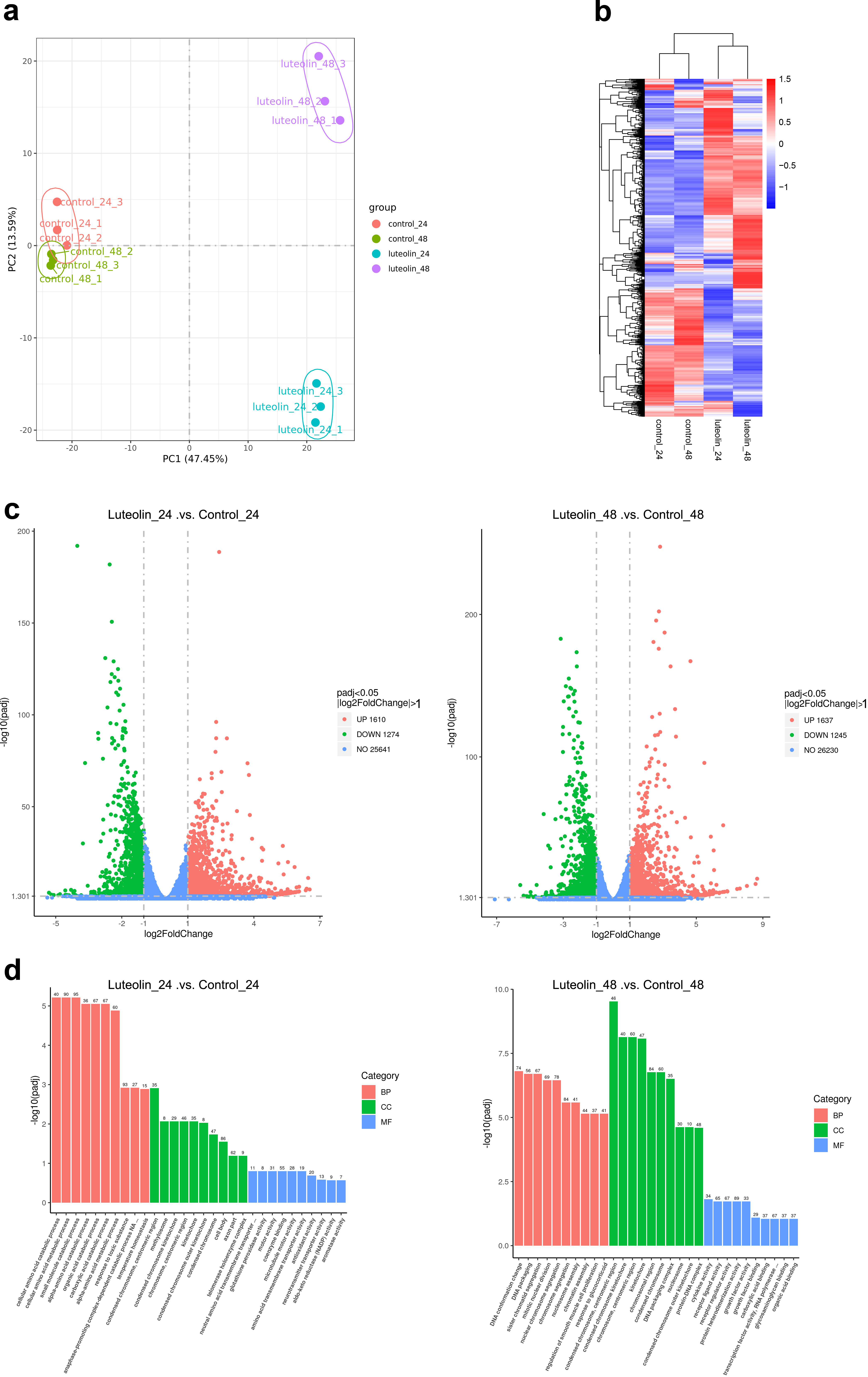


**Supplemental Figure 6.** Differentially expressed genes (DEGs) between luteolin-treated cells and untreated cells at the mRNA level. (a) Principal component analysis from 3 replicates of the Control_48 group and Control_24 group, the Luteolin_24 group and Control_24 group, the Luteolin_48 group and Control_48 group, and the Luteolin_48 group and Luteolin_24 group. The distance between the dots represents the overall expression differences of samples. (b) Heatmaps of gene expression levels of DEGs plotted as log_2_(FPKM+1) for the indicated samples. (c) Volcano plot showing the distribution trends of DEGs between the Control_48 group and Control_24 group, the Luteolin_24 group and Control_24 group, the Luteolin_48 group and Control_48 group, and the Luteolin_48 group and Luteolin_24 group. (d) GO enrichment of DEGs. The top 20 entries of biological process (BP), cellular component (CC), and molecular function (MF) are displayed with their gene number ranked from left to right based on ‑log10 (P_adj_).


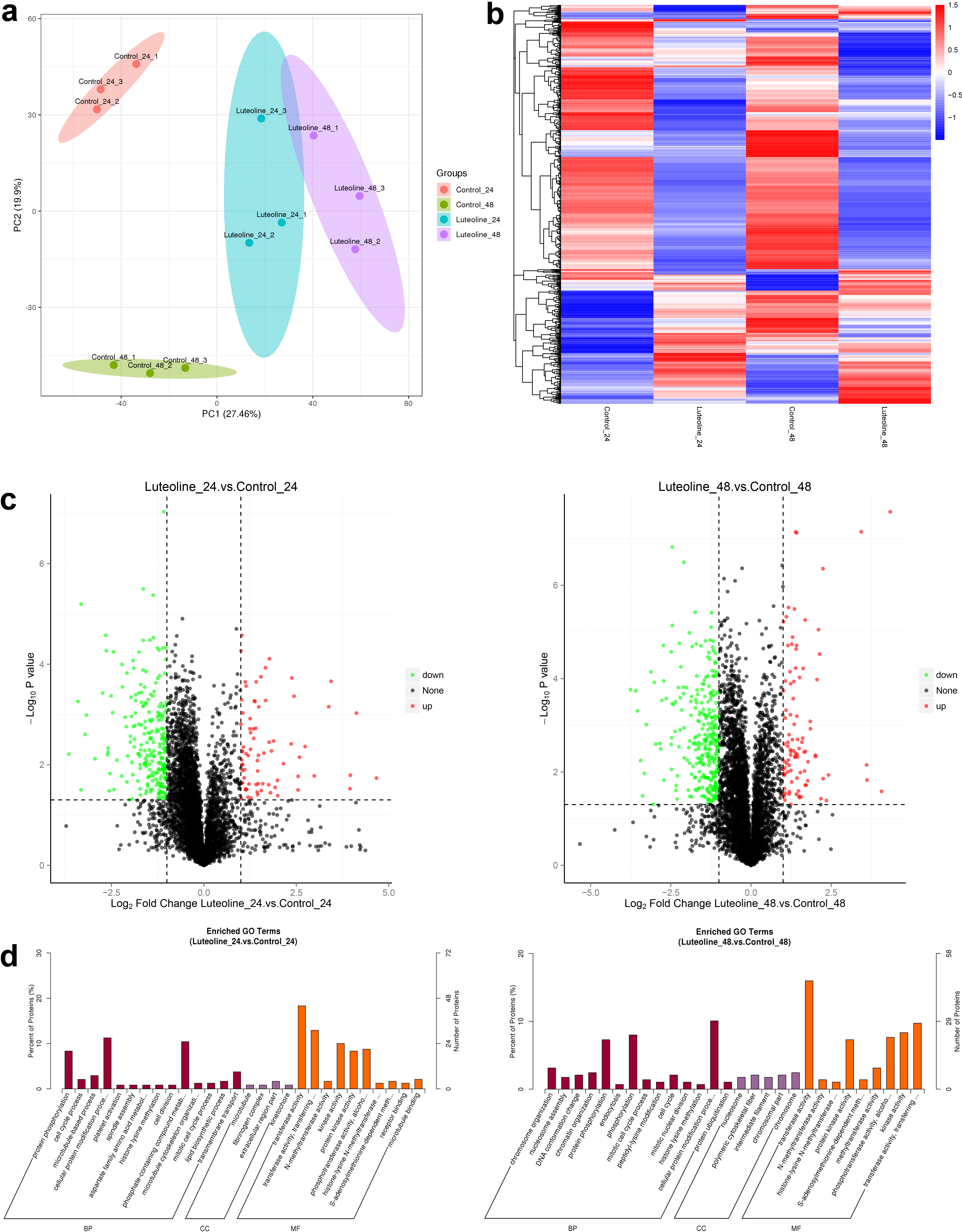


**Supplemental Figure 7. Differentially expressed genes (DEGs) between luteolin-treated cells and untreated cells at protein level.** (a) Principal component analysis from 3 replicates of the Control_48 group and Control_24 group, the Luteolin_24 group and Control_24 group, the Luteolin_48 group and Control_48 group, and the Luteolin_48 group and Luteolin_24 group. The distance between the dots represents the overall expression differences of samples. (b) Heatmaps of gene expression levels of DEGs plotted as log_2_(FPKM+1) for the indicated samples. (c) Volcano plot showing the distribution trends of DEGs between the Control_48 group and Control_24 group, the Luteolin_24 group and Control_24 group, the Luteolin_48 group and Control_48 group, and the Luteolin_48 group and Luteolin_24 group. (d) GO enrichment of DEGs. The top 20 entries of biological process (BP), cellular component (CC), and molecular function (MF) are displayed with their gene number ranked from left to right based on -log10 (P_value_).


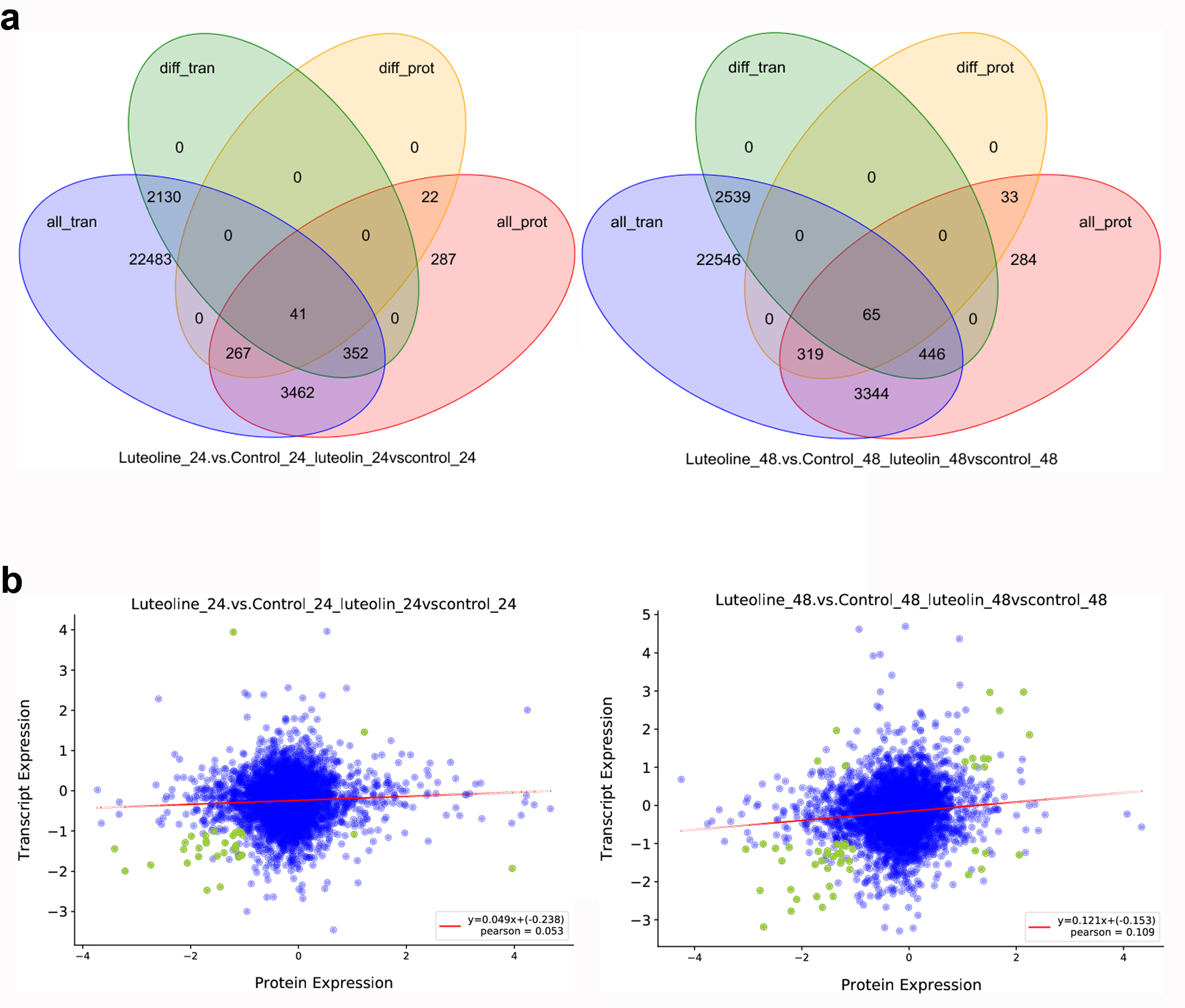


**Supplemental Figure 8.** (a) Venn diagram of all differentially transcribed genes and differentially translated proteins. In the Venn diagram, blue (all_tran) represents all genes obtained from the transcriptome, green (diff_tran) represents differentially expressed genes identified by the transcriptome, red (all_prot) represents all proteins identified by the proteome, and orange (diff_prot) represents differentially expressed proteins identified by the proteome. (b) Correlation analysis of transcriptome and proteome expression levels. In the correlation analysis graph, green dots represent significant differentially expressed proteins and blue dots represent nonsignificant differentially expressed proteins. The abscissa represents differentially translated proteins (log_2_FC) and the ordinate represents differentially transcribed genes (log_2_FC).

**References**

Liu JF, Ma Y, Wang Y, Du ZY, Shen JK, Peng HL. Reduction of lipid accumulation in HepG2 cells by luteolin is associated with activation of AMPK and mitigation of oxidative stress. Phytother. Res. 2011; 25 (4): 588–596.

Xu H, Yang T, Liu X, Tian Y, Chen X, Yuan R, Su S, Lin X, Du G. Luteolin synergizes the antitumor effects of 5-fluorouracil against human hepatocellular carcinoma cells through apoptosis induction and metabolism. Life Sci. 2016; 144: 138-47.

Yee SB, Choi HJ, Chung SW, Park DH, Sung B, Chung HY, Kim ND. Growth inhibition of luteolin on HepG2 cells is induced via p53 and Fas/Fas-ligand besides the TGF-β pathway. Int. J. Oncol. 2015; 47(2): 747–754.

Hwang JT, Park OJ, Lee YK, Sung MJ, Hur HJ, Kim MS, Ha JH, Kwon DY. Anti-tumor effect of luteolin is accompanied by AMP-activated protein kinase and nuclear factor-κB modulation in HepG2 hepatocarcinoma cells. Int. J. Mol. Med. 2011; 28(1): 25–31.

Androutsopoulos VP, Spandidos DA. The flavonoids diosmetin and luteolin exert synergistic cytostatic effects in human hepatoma HepG2 cells via CYP1A-catalyzed metabolism, activation of JNK and ERK and P53/P21 up-regulation. Journal of Nutritional Biochemistry. 2013; 24: 496-504.

Niu JX, Guo HP, Gan HM, Bao LD, Ren JJ. Effect of luteolin on gene expression in mouse H22 hepatoma cells. Genet. Mol. Res. 2015; 14(4): 14448–14456.

Ding S, Hu A, Hu Y, Ma J, Weng P, Dai J. Anti-hepatoma cells function of luteolin through inducing apoptosis and cell cycle arrest. Tumour Biol. 2014; 35(4): 3053–3060.

Selvendiran K, Koga H, Ueno T, Yoshida T, Maeyama M, Torimura T, Yano H, Kojiro M, Sata M. Luteolin promotes degradation in signal transducer and activator of transcription 3 in human hepatoma cells: an implication for the antitumor potential of flavonoids. Cancer Res. 2006; 66(9): 4826–4834.

Yoo DR, Jang YH, Jeon YK, Kim JY, Jeon W, Choi YJ, Nam MJ. Proteomic identification of anti-cancer proteins in luteolin-treated human hepatoma Huh-7 cells. Cancer Letters. 2009; 282: 48-54.
